# Enhancing 3D Printing Performance and Product Quality Through the Valorization of Food By‐Products and Waste

**DOI:** 10.1111/1541-4337.70267

**Published:** 2025-09-14

**Authors:** Mohammed A. Bareen, Derossi Antonio, Maria G. Corradini, Caporizzi Rossella, D'Incecco Paolo, Sindaco Marta, Severini Carla

**Affiliations:** ^1^ Department of Agriculture, Food, Natural Resources and Engineering University of Foggia Napoli Italy; ^2^ Department of Food Science and Arrell Food Institute University of Guelph Guelph Ontario Canada; ^3^ Department of Food, Environmental and Nutritional Sciences (DeFENS) Università degli Studi di Milano Milan Italy

**Keywords:** circular economy, food by‐products, 3D printing parameters, 3D printing techniques, secondary products

## Abstract

This review systematically explores the emerging use of food by‐stream materials in 3D printing (3DP) applications, addressing the pressing need for sustainable resource utilization in the food sector. The review evaluates the potential of food by‐products and waste, ranging from agricultural, animal, marine, microbial fermented biomass, and filamentous‐fungi by‐products to food consumption waste, for integration into 3DP techniques like fused deposition modeling, paste extrusion, direct ink writing, and selective laser sintering. Utilizing the PRISMA 2020 framework, a comprehensive literature analysis identified 80 relevant studies, categorized by material type and application. The findings indicate that plant‐based by‐stream materials encompassing sources like vegetable residues, fruit peels, nut and bean shells, and grain husks, dominate current 3DP research. These materials support biocomposite advancements across various fields, with notable applications in food‐safe packaging, biomedical scaffolds, nutritious snacks, and sustainable construction materials. Several studies highlight significant improvements in mechanical strength, such as tensile and compressive performance, alongside enhanced biodegradability of nonedible printed products and nutrient content in edible printed products. Key process parameters, including extrusion speed, nozzle temperature, and layer thickness, have been optimized to accommodate the unique properties of these food by‐stream materials, ensuring printing fidelity, smooth extrusion, and structural integrity, thereby maximizing their potential across diverse 3DP techniques and applications. This review highlights 3DP as a transformative approach in resource recovery, demonstrating how incorporating food by‐stream materials aligns with circular economy goals by reducing waste and enabling eco‐friendly production. By advancing customizable, nutrient‐dense, and sustainable products, 3DP of food by‐stream materials holds significant promise for addressing global food security and sustainability challenges.

## 1 Introduction

The integration of information and communication technology (ICT) into production processes is driving a profound reshaping of industrial manufacturing in the emerging digital world. A key enabling technology with high impact in this digital revolution is additive manufacturing (AM), or three‐dimensional (3D) printing. This technology offers unprecedented precision and ability to customize products, which positions it as a critical enabler of cyber‐physical systems (CPS) (Huang et al. 2015). The ability of producing intricate, tailored designs not only extends the manufacturing capabilities to limits previously unimaginable but also supports broader industrial goals by aligning production processes with the needs for a more efficient and sustainable food supply chain. Furthermore, 3D printing (3DP) plays a pivotal role in advancing the principles of the circular economy (CE), a framework designed to be inherently regenerative and restorative (Rosa et al. 2019). CE aims to reduce waste by optimizing the design of materials, products, systems, and business models, creating a closed‐loop system for sustainable resource use. This approach is gaining momentum in the agri‐food sector, with companies (Danone 2020; Nestlé 2020; PepsiCo 2020; Unilever 2020) and governments (Castillo‐Díaz et al. 2023; Hoehn et al. 2021; Kumar et al. 2023) actively pursuing and enacting a CE framework as a strategy for sustainable development. Danone's regenerative agriculture roadmap (DAR) requires that 30% of its priority ingredients be sourced from farms formally transitioning to regenerative practices by 2025, while its packaging plan targets 100% reuse, recyclability, or compostability and a 30% reduction in virgin plastics by 2030 (Danone 2020). Nestlé’s net‑zero roadmap allocates CHF 3.2 billion (2021–2025) to develop low‑impact materials packaging aiming to halve absolute greenhouse‑gas emissions (GHG) by 2030 and attain net‑zero by 2050 (Nestlé 2020). In addition, Unilever's financed €1 billion for waste‑free world (WFW) strategy aimed at reshaping all packaging into reusable, recyclable, or compostable materials and to a 50% cut in virgin plastics; the strategy also involved the potential use of 3D‑printed refill systems (Unilever 2020). At the governmental level, the European Union couples its CE action plan with the Green Deal, Farm‑to‑Fork, Biodiversity Strategies, and Sustainable Development Goals (SDGs), dedicating more than 35% of the re‑formed CAP budget to eco‑schemes that reward circular practices (Castillo‐Díaz et al. 2023).

The global food industry generates substantial residual materials, driving growing interest in repurposing these resources for sustainable applications. In this review, we use the term “food by‐stream (FBS) materials” to denote all residual outputs, whether processing by‐products or postconsumer food waste, that can be recovered and valorized via 3DP. These materials encompass agricultural residues (such as stalks, leaves, and husks), animal by‐products (including bones, blood, and feathers), food processing outputs (such as fruit and vegetable peels, seeds, pulp, whey, and spent grains), and waste from food consumption (like food scraps, leftovers, and expired products). According to the FAO (2019), food waste typically arises at the consumption stage, driven by consumer behavior and decisions from retailers and food services, leading to a reduction in both food quality and quantity. Approximately one‑third of the food produced for human consumption, about 1.3 Gt/yr is lost or wasted (Nordin et al. 2020). Losses are uneven across industries. Beverage plants discard the most, about 26% of the total. Dairy facilities follow at 21%. Fruit and vegetable processing, cereal milling, and meat preservation account for 14.8%, 12.9%, and 8%, respectively. Lower shares come from edible oil production (3.9%) and fish freezing or curing (0.4%); other activities make up the remaining 12.7% (Baiano 2014). Similarly, discarded food is not confined to one region. Developed nations (≈ 1.4 billion people) and developing nations (≈ 6.2 billion) together waste about 670 million tons of edible products each year (Amicarelli and Bux 2021; UNEP 2024). Meanwhile, nearly three billion people cannot afford a healthy diet, and 690–829 million suffer chronic hunger (FAO 2019).This waste is a major contributor to environmental challenges, including carbon emissions and resource depletion. On the other hand, food by‐products are secondary materials produced during food manufacturing, such as peels, seeds, and pulp. While not destined for direct consumption, these by‐products have significant potential for reuse.

**FIGURE 1:**
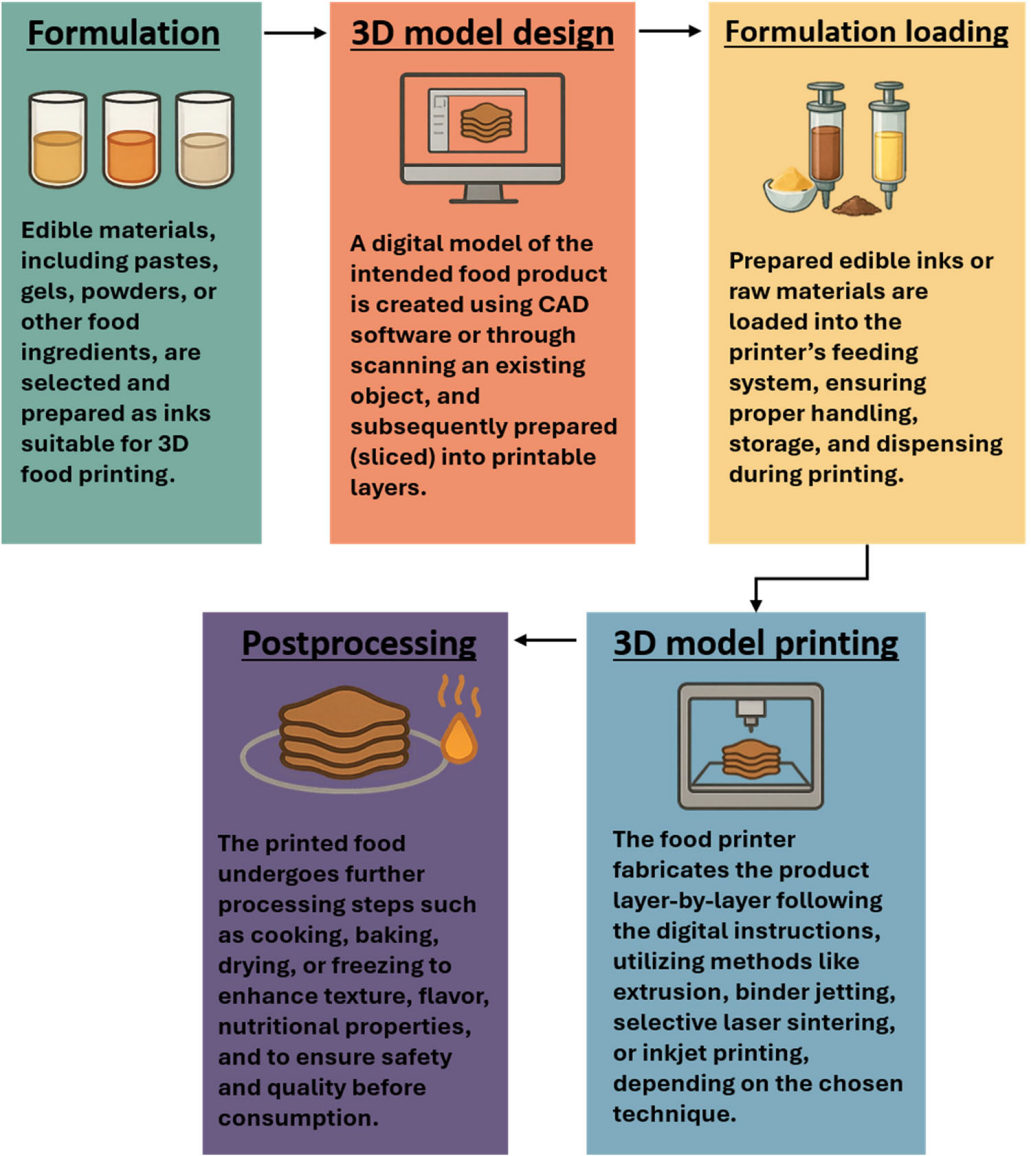
Overview of the general workflow involved in 3D food printing.

As the world faces the dual challenges of population growth and increasing demand for nutritious food, the food industry finds itself at a pivotal moment with the potential of redefining its role in food security (Adams et al. 2020; Geissdoerfer et al. 2017). 3DP offers significant advantages in addressing the challenges posed by traditional mass food production methods, including adaptability, nutritional precision, sustainability, local production, and supply chain resilience. The capacity of 3DP to innovate within food production processes dovetails with the goals of CPS, ensuring that food manufacturing can adapt to these changing requirements. The food production by‐stream materials are rich in nutrients and bioactive compounds, offering vast potential for repurposing in innovative applications like 3D food printing (3DFP). The integration of FBS materials into 3DFP not only supports sustainable food production but also aligns with CE principles by producing eco‐friendly, nutritious products. This innovative approach aligns with the principles of CE and reinforces sustainability within the agri‐food sector, minimizing its environmental footprint (Hassoun et al. 2023; Hooi Chuan Wong et al. 2022; Tyupova and Harasym 2024; Yu and Wong 2023). One of the key advantages of 3DFP lies in its capacity to tailor food structure and composition to meet specific nutritional requirements and personal dietary preferences (Figure 1). Nutrient‐rich powders derived from fruit and vegetable processing residues can be incorporated into printable matrices, thereby enhancing the nutritional profile of the final printed product (Tomašević et al. 2021). Because printable inks can be prepared from peels, pomace, or other residual fractions thereby making food more appealing to the consumer while also minimizing waste (Kadival et al. 2023). Converting these residues into extrusion‑ready pastes can trim ingredient‐associated costs by roughly 20%–40% (Kadival et al. 2023). Once printing begins, the process runs at set, comparatively mild temperatures. Energy consumption estimates for 3DFP show electricity demands up to 30% lower than those of baking, frying, or conventional extrusion, which translates into overall energy savings of about 40%. Localized, on‑demand fabrication also shortens supply chains: transport outlays fall by 25%–35% when products are printed where they are consumed (Kadival et al. 2023). A restaurant‑scale trial demonstrated that producing plant‑based “meat” on site with a desktop printer cut logistics costs by 30% relative to receiving frozen patties from a central plant (T. Wang et al. 2022). As 3DFP remains an emerging technology, there are currently no specific regulations that directly govern its processes and applications. Additionally, scholarly literature addressing regulatory frameworks for this field is still limited. Internationally, dedicated legal frameworks for 3D‐printed foods are still nascent, with most jurisdictions applying existing food safety laws to this novel manufacturing method (Baharuddin et al. 2023). In the European Union, 3D‐printed edibles are generally treated as novel foods under Regulation (EU) 2015/2283, necessitating premarket safety assessment and compliance with all usual food hygiene and labeling requirements (Baiano 2020; The European Parliament and the Council of the European Union 2015). Global standard‐setting bodies such as codex Alimentarius commission and FAO have indicated that potential hazards of 3D‐printed foods can be managed with established food safety risk assessment protocols (e.g., HACCP) and good hygienic practices (FAO 2023). It is noteworthy that, although the technology is still in its formative stages, regulatory frameworks may serve to facilitate innovation rather than restrict it. Nonetheless, the market for 3D‐printed food products is evolving, with significant interest from sustainability‐focused manufacturers and niche consumer segments. In the United States, the National Science Foundation has funded multi‐institutional partnerships to adapt extrusion and binder‐jet technologies for nutritionally customizable snacks and desserts (Nelson 2023). NASA's collaboration with BeeHex has yielded the Chef3D platform qualified for operation aboard the International Space Station that prints rehydratable pizza and other shelf‐stable meal components (NASA Spinoff 2019). At the supranational level, the EU's Bio‐Based Industries Joint Undertaking (BBI JU) supports the BARBARA project, which converts citrus peels, almond shells, and corn by‐products into bioplastic filaments for 3DP, illustrating cross‐sector valorization of agri‐food residues (European Commission 2019). These initiatives illustrate efforts by governmental offices and the food industry to commercialize 3D‐printed foods and embed them within broader agri‐food and healthcare strategies. Several studies have explored the use of 3DP for valorizing numerous agricultural wastes and by‐products, along with food wastes and by‐products. Rahman et al. (2023) provided a detailed analysis regarding the potential of agricultural‐derived biowaste in AM, particularly the use of rice husks, wheat straw, and sugarcane bagasse (SCB). Romani et al. (2023) focused on the valorization of biomass in the context of producing novel materials for 3DP applications. Additionally, the potential conversion of agri‐food waste streams into edible and inedible products through 3DP has been examined by Yoha and Moses (2023). Lastly, the use of food by‐products in 3DP has been discussed, primarily in terms of sustainability and material recovery (Yu and Wong 2023). However, none of these studies analyzed how the use of food by‐products and food waste might improve 3DP applications. This gap in the literature underlines the need for a more targeted survey and analysis that not only covers how food by‐products and food waste can be repurposed for sustainability but also clarifies their role in improving the performance of printable materials as well as the nutritional and sensory quality of 3D‐printed products. This review critically analyzes existing research to illustrate how and to what extent the incorporation of food by‐products and waste in 3DP can foster sustainable manufacturing practices. The primary aims are to: (1) assess the potential of food by‐products and waste—originating from harvest, postharvest, processing, and consumption stages—for resource recovery and enhanced sustainability; (2) examine advancements in the development of 3DP inks and formulations derived from food by‐products and/or recovered materials; (3) demonstrate how food by‐products and waste can contribute to enhance the performance of 3DP technology and the properties of 3D‐printed food, ultimately advancing customized and sustainable food production; and (4) assess the potential of such unmissable resources to improve material functionality for specific applications, for example, packaging films, biodegradable materials for food and nonfood use.

## 2 Material and Methods

This review provides a thorough evaluation of the literature on food wastes and by‐products for various 3DP methods. The main research questions (RQ) addressed herein are:

RQ1: What is the current state‐of‐the‐art 3DP using FBS materials, and what types of materials are primarily utilized?

RQ2: What are the commonly used 3DP methods for novel materials incorporating FBS materials, and what are the main printing parameters affected by the addition of upcycled components?

RQ3: What are the potential applications and constraints of using FBS materials for 3DP, with a particular focus on improving ink and final product properties for food and other industrial applications such as packaging, sustainable construction material, and the biomedical uses?

The methodology used involved a comprehensive analysis of the existing literature to assess the current prospects of 3DP in FBS materials utilization across various aspects, including improving printability, mechanical properties, nutritional profile, sensory characteristics, and biodegradability. A comprehensive literature review following the guidelines set forth by the PRISMA 2020 systematic review framework (Sarkis‐Onofre et al. 2021) was conducted. The adoption of PRISMA 2020 provides substantial benefits for authors, editors, and peer reviewers engaged in systematic reviews. It enables readers to critically assess the validity of the methodologies used, thereby reinforcing the credibility of the findings. The primary objective of the PRISMA framework is to enhance the thoroughness, precision, and openness of systematic reviews. PRISMA includes a checklist and a flow chart that outline the processes for searching, identifying, screening, and analyzing literature, ensuring that the gathered information is comprehensive. Table 1 details the eligibility criteria, databases searched, and specific query strings employed to select the articles reviewed in this study. Notably, previous reviews and meta‐analysis articles were purposely excluded, as studies with an experimental component related to various AM methods were primarily targeted.

**TABLE 1:** Criteria for eligibility and search library and binary query strings used.

Eligibility criteria	Search database	Query string
To be accessible from the library To use 3D printing methods for product manufacturing To include food by‐products or food by‐product utilization To focus on experiments, characterization or applications No review articles To be published in English	Scopus and ScienceDirect	(“additive manufactur*” OR “3D‐printing” OR “rapid prototyp*”) AND (“food waste” OR “food by‐product”)AND (“valorization” OR “value‐added” OR “waste recovery”)
	Web of Science	(((ALL=(additive manufacturing OR 3D‐printing OR rapid prototyping)) AND ALL = food by‐products OR food by‐product)) AND ALL = (Valorization OR value‐added OR waste recovery))

To maintain the review's focus on FBS materials in 3DP, studies dealing with chemical wastes or nonfood industrial residues were excluded, whereas those involving biowaste from food‐related aquatic sources (fish and algae) were retained. Studies that referenced 3DP but did not formulate printed materials incorporating food waste or by‐products were likewise excluded from the final dataset. Additionally, papers that lacked descriptions of AM methods, such as printer type, materials used, or newly developed materials, were not considered. The literature search was conducted using Scopus, ScienceDirect, and Web of Science databases.

**FIGURE 2:**
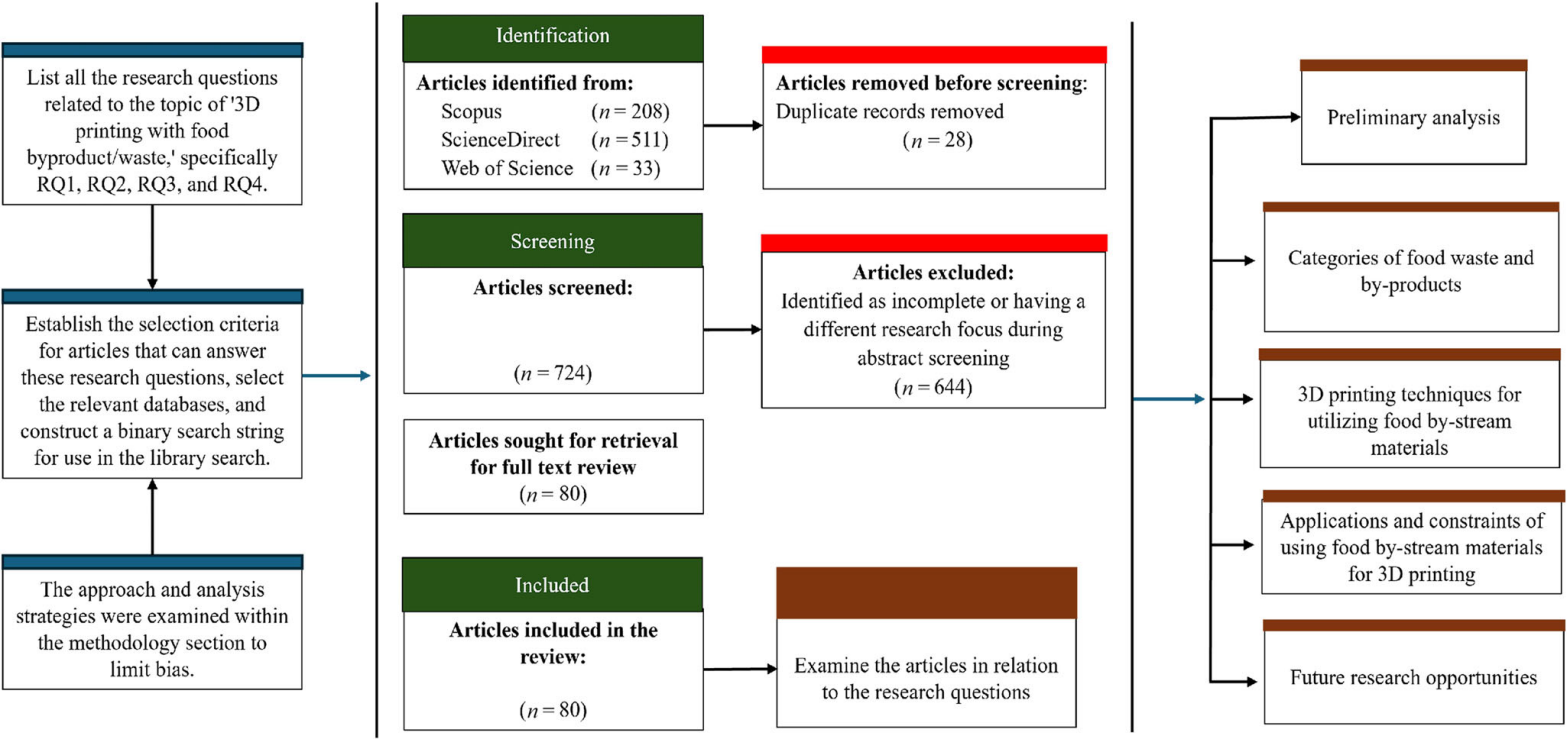
PRISMA flow chart demonstrating the selection process applied during the literature review presented in this work.

The search results were first filtered to exclude duplicates and unavailable articles. To select the data to be included in the bibliometric analysis, several prescreening procedures were performed by examining titles, abstracts, and keywords of the scientific documents. After this initial screening, a comprehensive textual analysis was conducted on the topics of food by‐products, 3D food methodologies, and innovative materials integrating food waste. A thorough analysis of the complete manuscripts was conducted, followed by two additional screening steps. First, any review papers and scientific documents lacking complete methodologies, such as conference papers, were excluded. Second, research papers were excluded if they merely referenced 3DP without actually utilizing it. This was performed to guarantee that every retained study provides sufficient information for replicability of findings and the comprehensive availability of methodological details. Furthermore, during data extraction, data columns corresponding to article classifications, hyperlinks, important terms, material requirements, vendors, origin, 3DP techniques, potential applications, constraints, and future research prospects were identified (Figure 2). The data were retrieved from full texts, selected websites, and source sites. In certain situations, the main intended use was for comparison with data in other articles, such as printing parameters. Values for all key printing parameters were converted to consistent conventional units—viscosity (Pa·s), extrusion/print speed (mm/s), nozzle diameter and layer height (mm), extrusion pressure (kPa), and temperature (°C)—to allow direct comparison across studies. While the core focus is edible products, selected nonfood cases are summarized to illustrate how the same 3DP strategies open further value‐channels, packaging, biomedical, and construction that ultimately strengthen the economic viability of up‐cycling food‐stream materials. While preparing this work, the authors used QuillBot/ChatGPT to paraphrase and check the sentences grammatically. After using this tool/service, the authors reviewed and edited the content as needed and took full responsibility for the content of this article.

## 3 Results and Discussion

The screening process was applied to articles published in the past 10 years (2014–2024) and as outlined in the previous section identified a total of 80 research publications. This demonstrates a growing interest in the use of 3DP as tools to create sustainable and renewable products. This section presents the analysis of those 80 articles. Subsection 3.1 discusses state‐of‐the‐art research regarding the types of FBS materials for 3DP giving some insights on RQ1. Subsections 3.2–3.4 focus on analyzing commonly used 3DP methods incorporating FBS materials and their specifications (RQ2), potential applications of FBS materials in 3DFP (RQ3) and the future applications, limitations, and perspectives answering RQ3.

### 3.1 3D‐Printable Materials From FBS

The systematic review reveals a significant increase in the number of research articles on 3DP using FBS materials after 2020. Particularly, in 2021, 19 papers were published, greater than the total number of scientific documents published between 2016 and 2020. Then, by evaluating several key factors in the publications (e.g., materials utilized, source or origin of the materials, etc.) the FBS materials used in 3DFP were categorized into four groups. The categories include plant‐derived, animal‐ and marine‐sourced, biomass, and by‐products from fermentation and extraction. Plant‐based waste and by‐products was the largest category, accounting for 49% of the total articles. This was followed by articles on by‐products and waste from animal and marine sources, representing 25% of the total. Studies on biomass research accounted for 15% and by‐products from fermentation and extraction 11%. Plant‐based products, including grains, nuts, soybeans, and biomass represent 64% of the total research articles, underscoring the significant focus on plant‐based sources for 3DP using FBS materials. The selected articles demonstrate the considerable potential for resource recovery across various FBS through the use of 3DP. By leveraging these materials, several industries (e.g., food, pharmaceutical, construction) could reduce their reliance on virgin resources, and minimize waste and carbon footprints, thereby contributing to a more circular and sustainable economy. Table 2 lists the selected articles by FBS material types, product/high‐value resource from FBS, 3DP ink matrix, and weight percentage of food by‐stream material in the matrix.

**TABLE 2:** Selected 3D printing applications of food by‐stream materials, categorized by types.

Food processing waste category	3D‐printed product/application	Food by‐stream material	Combined matrix	Weight percentage used (wt%)	Ref.
Animal and marine processing	Bio‐welded brick composite using mycelium as bonding	Mollusk shells	Seashell biocomposite with *Pleurotus ostreatus* mycelium	80	Abdallah and Estévez (2023)
	Hydrogels for biomedical scaffolds	Chitosan from crustacean shells	Chitosan/hyaluronic acid polyelectrolyte complex	20	Braccini et al. (2024)
	Chitin‐based architecture for tissue engineering	Crayfish shells	Chitin extracted from crayfish shells, acrylic acid, polyethylene glycol diacrylate, and photoinitiator	6	Li, et al. (2023a)
	Biocomposite filament	Crab shell (*Callinectes sapidus*)	Polylactic acid mixed with 7% crab shell particles	7	Palaniyappan et al. 2024
	Biocomposite material for bone tissue engineering	Crab shell powder	Crab shell powder incorporated into poly(lactic acid)	1.5	Yang et al. (2023)
	Chitosan scaffold for wastewater remediation	Chitosan (derived from crab and other crustacean shells)	Chitosan combined with TiO_2_ nanoparticles	6	Bergamonti et al. (2019)
	Biocomposites for biomedical applications	Calcined *Mediterranean* Seashell waste	PLA matrix reinforced with calcined seashell (CSh) powder and stearic acid	30	Gnanamani Sankaravel et al. (2022)
	Electroconductive ink for strain sensing applications	Prawn shells (chitosan extracted from prawn shells)	Chitosan mixed with carbon fibers and silk fibroin	27	Sanandiya et al. (2024)
	Protein‐enriched dough	Porcine plasma protein	Doughs with porcine plasma protein, soy protein isolate, pea protein concentrate, and glycerol	42.5–47.5	Álvarez‐Castillo et al. (2021)
	Salmon skin gels	Salmon skin	Salmon skin gelatin gelled at various concentrations	2–14	Carvajal‐Mena et al. (2022)
	3D‐printed surimi	Cod by‐products	Surimi made from cod by‐products	< 99	Gudjónsdóttir et al. (2019)
	Biodegradable composite filament	Anchovy fish bone powder	Anchovy fish bone powder mixed with polylactic acid (PLA) or Mater‐Bi	10–20	Scaffaro et al. (2022)
	3D‐printed PCL/nano‐hydroxyapatite scaffolds	Cuttlefish bones, mussel shells, chicken eggshells	PCL mixed with nano‐hydroxyapatite synthesized from cuttlefish bones, mussel shells, and eggshells	13–15	Cestari et al. (2021)
	3D‐printed composite filament	Fish scale‐derived hydroxyapatite and eggshell	PLA mixed with fish scale‐derived hydroxyapatite (FHAp) and calcined eggshell powder (EGS)	3–9	Wu et al. (2021)
	Ribose‐crosslinked gelatin products	Gelatin	Gelatin, crosslinked ribose and glycerol	2.5–5	Stevenson et al. (2019)
	Cheddar cheese	Whey protein isolate (WPI)	Reduced‐fat processed cheese and WPI nanofibrils	0–40	D. Wang et al. (2023)
	Level 5 minced and moist—dysphagia food	Whey protein isolate (WPI)	Soybean oil, WPI, NaCl and Transglutaminase	12.73	Li et al. (2024)
	Konjac hybrid gel	Whey protein powder (WP)	Konjac flour, WP, Sodium bicarbonate	5–30	Du et al. (2021)
	3D‐printed edible food for dysphagia	Whey protein isolates (WPI)	Whey protein isolate nanofibrils, 4′,6‐diamidino‐2‐phenylindole (DAPI), *Lactiplantibacillus plantarum*	5	Zhang et al. (2024)
	Scaffolds for wound dressing applications	Whey Protein Concentrate (WPC)	Poly lactic acid, WPC, Dimethylformamide and Dichloromethane	25–50	Kayadurmus et al. (2024)
	Bio composite scaffolds with drug delivery capability	Whey protein isolate (WPI)	Polyvinyl alcohol, WPI, hydroxyapatite, gentamicin	10	Tut et al. (2022)
Plant‐derived product processing	Unique and appealing snacks	Imperfect broccoli and carrots	Broccoli and carrot powders with wheat flour, olive oil, and salt	25–75	Ahmadzadeh et al. (2024)
	3D‐printed edible food for dysphagia	Spinach stems and kale stalks	Spinach and kale purees with potato and corn starch	50	Pant et al. (2023)
	Photocurable formulation for VAT printing	Aloe vera peel	Microcrystalline cellulose with fish skin gelatin, polyethylene glycol diacrylate, sodium carbonate, hydrochloric acid and allylamine	5–33	Cabua et al. (2024)
	3D‐printed noodles	Potato peel	Whole wheat flour and potato peel powder	40	Muthurajan et al. (2021)
	Biocomposite filaments	Tomato stems	PLA with tomato stem powder and Joncryl	5–10	Pemas et al. (2024)
	Devices for contextual and efficient fertilizer release and Cu(II) ions capture	Tomato stems and leaves	PLA and Mater‐Bi mixed with tomato plant waste and NPK fertilizer	10 or 20	Scaffaro et al. (2024)
	Bioflavonoid‐rich snacks	Orange peel	Rheology‐modified inks containing orange peel waste, with additives for enhanced printability	—	Leo et al. (2022)
	Fiber‐enriched apricot gel snacks	Orange by‐products	Gelatin gels with apricot pulp (30‐70%) and orange by‐products, freeze‐dried	3.5–5.8	Molina‐Montero et al. (2023)
	Bioactive‐rich snacks	Orange peel	Orange peel powder with xanthan gum	20	Da Tan et al. (2023)
	Biodegradable food packaging casings	Banana peel	Banana peel powder with 1% (w/w) guar gum	99	Nida et al. (2023)
	Theophylline oral films with a narrow therapeutic index	Durian rind	Carboxymethyl cellulose (CMC) from durian rind with theophylline	8	Panraksa et al. (2023)
	3D‐printable ink for use in healthcare	Durian husk (mesocarp and exocarp)	Durian husk powder with xanthan gum	25	Tan et al. (2022)
	Biocomposite filaments	Tomato plant after tomato harvest	Mater‐Bi polymer with tomato plant waste	5–15	Scaffaro et al. (2023)
	Natural filler composite filaments	Cocoa bean shells	Recycled polypropylene with 5 wt% cocoa bean shell particles	5	Morales, et al. (2021a)
	Biocomposite filaments	Cocoa bean shells	Poly(ε‐caprolactone) (PCL) with micronized cocoa bean shell powder	10–50	Tran et al. (2017)
	3D‐printed biodegradable food packaging	Banana peel and sugarcane bagasse	Mixture of banana peel and sugarcane bagasse	<99	Nida et al. (2022)
	Bioplastic composites	Mixed food waste (mainly starch and cellulose fractions, after enzymatic hydrolysis)	PHB produced from *Bacillus mycoides* using hydrolyzed food by‐products mixed with PHB/PMMA	<25	Rofeal et al. (2023)
	Biocomposite filaments	Hydroxypropyl methylcellulose (HPMC) derived from mixed vegetable waste	PLA with HPMC	—	Jiang et al. (2020)
	Bioplastic films	Wheat gluten	Gluten‐based bioplastic with tyrosine (for photocrosslinking) and keratin	70–100	Alshehhi et al. (2024)
	Biocomposite filaments and pellets	Beer bagasse (spent brewers' grain)	PLA combined with beer bagasse and plasticizers	10	Carichino et al. (2023)
	Natural fiber composite filaments	Rice husk	Recycled polypropylene with rice husk	5–10	Morales, et al. (2021a)
	Biomass composite material for high quality sintered parts using SLS	Rice husk	Rice husk powder incorporated into co‐polyamide	10	Li, et al. (2023a)
	Photo‐curable fiber composites using SLA	Rice straw and wheat straw	Rice straw and wheat straw fibers, photo initiator, acrylic monomers, and oligomers	5	Romero‐Ocaña et al. (2022)
	Biocomposite filament	Corncob powder	HB and PLA matrix with corncob filler at 2%‐8% by weight	2–8	Ohaeri and Cree (2022)
	Corn starch and cellulose fibers composites for the medical and food applications	Corn husks (cellulose fibers extracted)	Cellulose fibers from corn husks, corn starch	10	Islam and Jiang (2022)
	Hemicellulose and lignin ink without purification, modification, or auxiliary additives	Corn cobs	Crude corn cob extract	27	Gokce Bahcegul et al. (2022)
	Biocomposite filaments	Buckwheat husk	Buckwheat husk and polylactic acid and thermoplastic starch	—	Andrzejewski et al. (2021)
	3D‐printed gastric floating drug delivery systems	Brewer's spent grain, spent coffee grounds, sesame cake, thermoplastic starch	Polylactic acid (PLA) blended with food waste	15	Wang et al. (2024)
	Visually appealing snacks with customized texture	Okara	Okara powder and water	<33	Lee et al. (2021)
	Biocomposite filaments	Pyrolyzed soy hulls biocarbon	Recycled high‐density polyethylene, recycled polypropylene and pyrolyzed soy hulls biocarbon	<20%	Maldonado‐García et al. (2021)
	Biocomposite filaments	Macadamia nutshells	Ground macadamia nutshell mixed with ABS plastic and maleic anhydride	19 or 29	Girdis et al. (2017)
	Feedstock for SLS	Walnut shell	Walnut shell powder with copolyester and micro‐additives	20–40	Sun et al. (2023), Yu et al. (2017, 2018, 2021)
	Biocomposite filaments	Walnut shell powder, eggshell powder	PLA combined with walnut shell powder, and eggshell powder	2.5	Lohar et al. (2022)
	Biocomposite filaments	Australian royal palm fiber	Acrylonitrile butadiene styrene with palm fiber	<20	Marton et al. (2022)
	Biocomposite filaments	Organosolv lignin extracted from oil palm empty fruit bunch fibers	Acrylonitrile butadiene styrene with organosolv lignin and graphene nanoplatelets	<15	Mohan et al. (2021)
By‐products from fermentation and extraction	Functional cookies	Grape pomace	Wheat flour was replaced with 4%, 6%, and 8% grape pomace	4–8	Jagadiswaran et al. (2021)
	Colored biocomposite filaments	Spent coffee grounds (SCGs)	PLA with micro/nano‐structured decolorized spent coffee grounds (MN‐DSCGs)	2–20	Li et al. (2021)
	Biomass‐filled polymers inks for pellet extrusion	SCGs	PLA with spent coffee grounds	5–10	Paramatti et al. (2024)
	Biopolymer ink for fused granular fabrication	SCGs	PLA, LDPE, or HDPE with SCGs	10	Romani et al. (2024)
	Mycelium‐based composite as self‐healing materials and natural glues	SCGs	Agar‐based ink, with coffee grounds, agar, and liquid mycelium culture	5	Soh et al. (2023)
	Cellulose/ soy protein‐based hydrogels	Cellulose‐containing residues from agar production	Soy protein isolate, agar residue, porcine gelatin, and glycerol	0–8	Uranga et al. (2023)
	Edible soy protein films	Grape seed, green tea	Soy protein isolate with grape seed extract or green tea extract	1–5	Ahmadzadeh et al. (2023)
	Biofillers containing composite powders for SLS	Corn germ meal by‐product (GTF) and wine production waste (WPL‐DH)	PBAT polymer mixed with GTF and WPL‐DH fillers	5–10 (for each filler)	Colucci et al. (2024)
	Biomass/PLA composites filaments	SCG and spent tea leaves (STL)	PLA with SCG or STL	40	Yu and Wong (2023)
	Biocomposite inks for architectural applications	SCG, eggshells, oyster shells	Hydrogel composed of xanthan gum, chlorella, cellulose, eggshell, charcoal, and SCG	<50	Choi and Yi (2024)
Biomass	Biomass–fungi composites	Biomass	Biomass fungi mix, wheat flour, and psyllium husk powder	50	Rahman et al. (2022)
	Biomass‐fungi composite	Biomass	Biomass fungi mix, wheat flour, and psyllium husk powder	20	Bhardwaj et al. (2021), Rahman et al. (2023)
	Fluorescent flax shives reinforced biocomposites	Flax shives	Flax shives incorporated into poly‐(butylene‐terephthalate)	10	Mayer‐Laigle et al. (2021)
	Biodegradable biocomposite filaments	Flax shives	Flax shives mixed with PLLA, PLLA/PBS, and PBAT matrices	10–30	Badouard et al. (2019)
	Biocomposite filaments	*Miscanthus* biocarbon	Poly(trimethylene terephthalate) with *Miscanthus* biocarbon	5–10	Diederichs et al. (2021)

#### 3.1.1 Animal and Marine Processing Waste and By‐Products

Materials of animal origin in the selected studies span various by‐products, including eggshells (Sankaravel et al. 2022), seashells (Abdallah and Estévez 2023), chitosan (Bergamonti et al. 2019; Braccini et al. 2024), chitin from crayfish (Li et al. 2023b), crab and prawn shells (Sanandiya et al. 2024; Yang et al. 2023), anchovy fishbone waste (Scaffaro et al. 2022), cod by‐products (Gudjónsdóttir et al. 2019), salmon skin gelatin (SSG) (Carvajal‐Mena et al. 2022), porcine plasma protein (PPP) (Álvarez‐Castillo et al. 2021), and whey protein (D. Wang et al. 2023; Zhang et al. 2024). These materials exhibit a relatively high content of valuable components such as calcium carbonate, chitin, collagen, and proteins, which provide mechanical reinforcement, enhance biocompatibility, carry antibacterial properties, and bestow functional characteristics such as viscoelasticity, gelling, or plasticizing effects. Given this, the 3D‐printable materials reinforced with these by‐products find applications in construction (Abdallah and Estévez 2023), biomedical engineering (Yang et al. 2023), tissue scaffolding (Sankaravel et al. 2022), food products (Álvarez‐Castillo et al. 2021), packaging (Gerna et al. 2025), and environmental remediation (Bergamonti et al. 2019).

##### 3.1.1.1 Nonfood‐Related Studies

Seashells, with a calcium carbonate (CaCO_3_) content of 95%–97%, when integrated into a biocomposite matrix reduced brick weight by 40% compared to clay bricks while maintaining dimensional stability (±1 mm). Similarly, chitosan, derived from the deacetylation of crustacean shells, was combined with TiO_2_ nanoparticles (20 nm) to develop a photocatalytic scaffold for wastewater treatment, achieving a 90% degradation of amoxicillin after 3 h of UV exposure. In another study, crayfish and crab shells with 25%–30% chitin and 31%–40% calcium carbonate content, respectively, enhanced the mechanical properties of polylactic acid (PLA), resulting in materials with 31% higher tensile strength and 47% more flexural strength. This reinforced material was suitable for biomedical applications, specifically bone scaffolds. Similarly, a PLA biocomposite fortified with 10% eggshell particles (with approximately 94% calcium carbonate), had a significantly higher compressive strength (94.9%) than pure PLA, while maintaining a 40% porosity, making it a viable material for bone tissue engineering. Another example of the use of animal by‐products to enhance rheological and mechanical properties was the incorporation of 13%–15% nano‐hydroxyapatite (nHA) from cuttlefish bones into polycaprolactone (PCL) composites, which resulted in significant improvements in scaffolds for bone regeneration, enhancing both compressive strength and porosity—key factors for bone growth (Cestari et al. 2021).

##### 3.1.1.2 Food‐Related Studies

**FIGURE 3:**
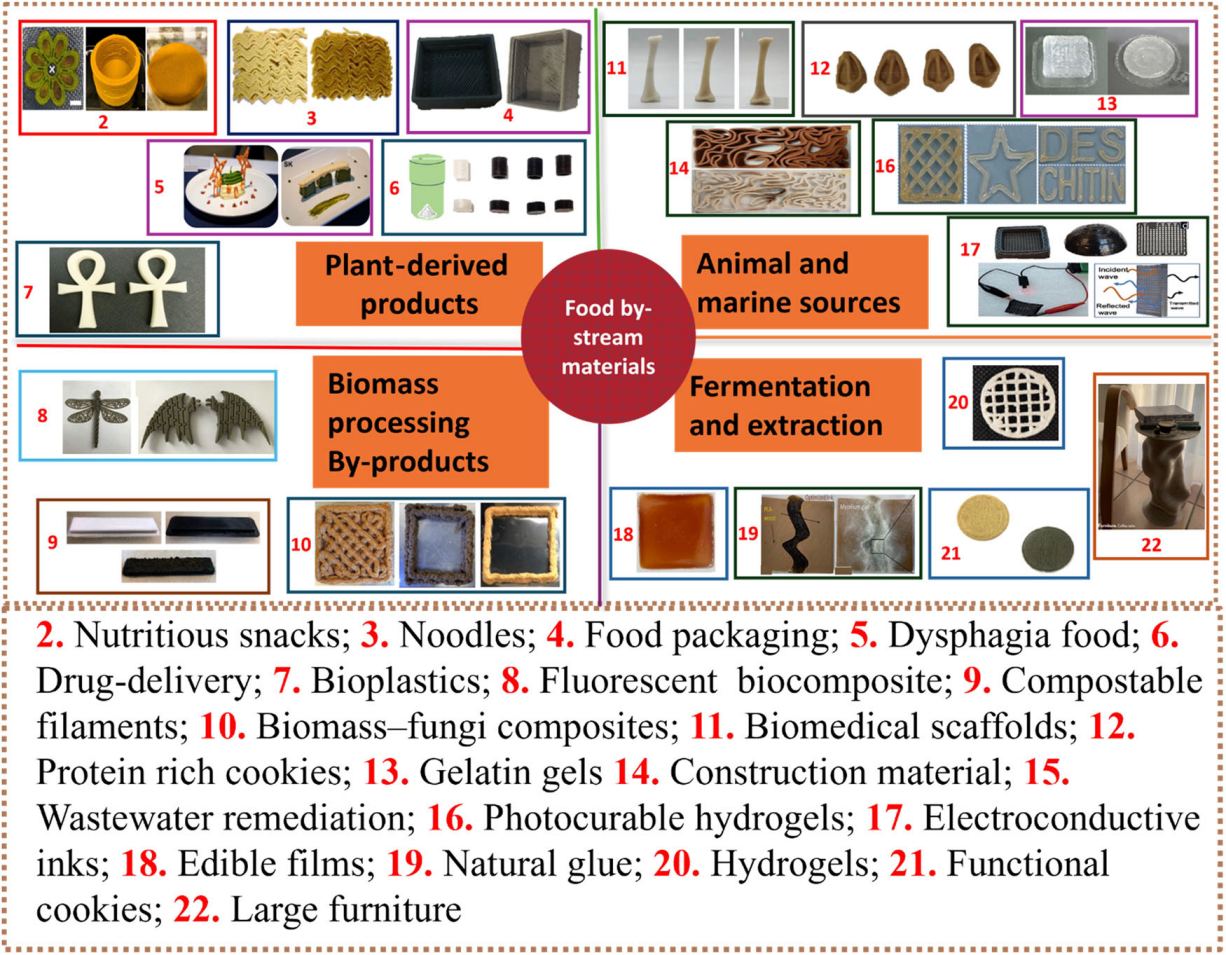
Food production by‐stream materials, classified based on origin, and applied to 3D printing, supporting sustainable valorization in plant, animal, marine, and industrial sectors. Image 2 is adapted from Da Tan et al. (2023), Leo et al. (2022), and Molina‐Montero et al. (2023); Image 3 is adapted from Muthurajan et al. (2021); Image 4 is adapted from Nida et al. (2022, 2023); Image 5 is adapted from Pant et al. (2023); Image 6 is adapted from L. Wang et al. (2024); Image 7 is adapted from Rofeal et al. (2023); Image 8 is adapted from Mayer‐Laigle et al. (2021); Image 9 is adapted from Diederichs et al. (2021); Image 10 is adapted from Bhardwaj et al. (2021) and Rahman et al. (2022, 2023); Image 11 is adapted from Yang et al. (2023); Image 12 is adapted from Álvarez‐Castillo et al. (2021); Image 13 is adapted from Carvajal‐Mena et al. (2022); Image 14 is adapted from Abdallah and Estévez (2023); Image 16 is adapted from Li et al. (2023a); Image 17 is adapted from Sanandiya et al. (2024); Image 18 is adapted from Ahmadzadeh et al. (2023); Image 19 is adapted from Soh et al. (2023); Image 20 is adapted from Uranga et al. (2023); Image 21 is adapted from Jagadiswaran et al. (2021); and Image 22 is adapted from Paramatti et al. (2024).

PPP, a high‐protein (74 wt%) by‐product from slaughterhouses, was combined with glycerol acting as a plasticizer to create dough‐like inks with improved rheological properties for 3DP applications (Figure 3). The innovative dough showed excellent printability and shape retention during printing, producing 3D‐printed snacks high in protein (45%–47.5%) (Álvarez‐Castillo et al. 2021). Salmon skin by‐products were utilized as a gelling agent due to their high content in collagen (approximately 75%) and gelatin. The salmon skin printable material, at an optimal concentration of 8%, was used to improve gelatin flow and dimensional stability of gelatin during layer‐by‐layer deposition by endowing the ink with shear‐thinning behavior and viscoelastic properties (Carvajal‐Mena et al. 2022). Another interesting example is cod by‐products, which, despite their low economic value, is a good protein source. The cod by‐products were transformed into surimi paste, using either conventional washing or the pH‐shift method, and salt concentration was modulated to optimize the 3DP process and improve gelling properties. This last application focused on producing 3D‐printed surimi products for consumer use, with enhanced water retention and structural integrity after cooking (Gudjónsdóttir et al. 2019). The use of dairy by‐products as ink for 3DP appears to be a relatively unexplored area of research. This is likely due to the fact that dairy by‐products are already efficiently reused within the dairy chain for the production of various food products, such as ricotta cheese, whey protein isolate (WPI), lactose, sport supplements and fermented or powdered buttermilk, etc. Among dairy by‐products, the whey deriving from cheesemaking is undoubtedly the most extensively utilized matrix, due to its abundance and low cost. The nutritional and functional properties of whey proteins, such as alpha‐lactalbumin and beta‐lactoglobulin, have led to the exploration of this by‐product for potential applications in 3DP, particularly in the form of WPI (Li et al. 2024; Wang et al. 2023). The primary applications of WPI in 3DP are within the food industry, particularly for utilizing the texturing properties of whey proteins, which can form a network structure when heated in mixed gel systems. This enhanced gel texture, in turn, improves the structural properties of the printed objects and in particular their self‐standing ability. Du et al. (2021) demonstrated that incorporating 20% whey proteins into a konjac flour mixed gel creates an optimal formulation for 3DP. In contrast, the addition of 25%–30% whey proteins, while providing sufficient structural support, can lead to inconsistencies in the printing process if there is a lack of adequate balance between printing speed and extrusion rate (i.e., the amount of material deposited per unit of time) which affect the printing fidelity. Denatured whey proteins, in the form of nanofibrils, have also been used to enhance the emulsifying, thickening, and gelling properties in 3D‐printed Cheddar cheese (Wang et al. 2023). This improvement was attributed to their ability to retain both water and fat during the printing process which prevented phase separation and oiling‐off during processing. Whey protein nanofibrils (WPNFs) were also utilized as a carrier and protective matrix for probiotic bacteria, enhancing their survival in 3D‐printed dysphagia‐friendly foods during gastrointestinal digestion (Zhang et al. 2024). Another application of WPI in nonfood 3DP is its use as a base biomaterial for medical implants (Kayadurmus et al. 2024), as well as a scaffold with drug delivery capabilities for bone tissue engineering (Tut et al. 2022).

#### 3.1.2 Plant‐Derived Processing Waste and By‐Products

The range of plant by‐products reported in the selected articles spans various sources, including broccoli and carrots (Ahmadzadeh et al. 2024), spinach stems and kale stalks (Pant et al. 2023), tomato stems and leaves (Pemas et al. 2024; Scaffaro et al. 2024), durian rinds and husks (Panraksa et al. 2023; Tan et al. 2022), and peels of aloe vera (Cabua et al. 2024), potatoes (Muthurajan et al. 2021), oranges (Da Tan et al. 2023; Leo et al. 2022; Molina‐Montero et al. 2023), and bananas (Nida et al. 2023). These materials contain significant quantities of bioactive compounds, fiber, cellulose, and starch, contributing to the nutritional and technological properties of printable materials.

##### 3.1.2.1 Food‐Related Studies

For example, fiber‐rich powders from broccoli and carrot residues enhance snack texture, layer resolution, and overall printability (Ahmadzadeh et al. 2024), while antioxidant‐ and fiber‐dense orange‐peel powders added to snack and apricot‐gel matrices simultaneously boost nutritional value and deliver smoother extrusion with better dimensional stability, together underscoring the versatility of plant‐processing wastes in 3DP (Da Tan et al. 2023; Leo et al. 2022; Molina‐Montero et al. 2023). Similarly, potato peels rich in starch and fiber were used to enhance the structural stability and the nutritional properties of innovative 3D‐printed noodles (Muthurajan et al. 2021) (Figure 3). Furthermore, okara, a soybean processing by‐product, has also been used to produce 3D‐printed food products with a high protein content of up to 28%, offering a nutritionally dense and structurally stable solution for personalized food products (Lee et al. 2021).

##### 3.1.2.2 Nonfood‐Related Studies

Tomato stems and leaves with high density in lignocellulosic material were processed into 3D‐printed biocomposites that acted as fertilizers and copper ion absorbers, showcasing their dual role in environmental applications and waste management (Scaffaro et al. 2024). Furthermore, durian rind and husk with a high cellulose content, were processed into printable films and mesh structures for biodegradable packaging and healthcare applications (Panraksa et al. 2023; Tan et al. 2022). Talline cellulose (MCC) from aloe vera peels allowed developing biodegradable 3D‐printed hydrogels, by fostering extensive cross‐linking and mechanical strength (Cabua et al. 2024). All these plant‐derived and soybean waste applications illustrate the potential for creating sustainable materials with tailored functional properties (e.g., mechanical, nutritional) that could drive eco‐friendly advancements in packaging, food production, and even in nonfood‐related industries, such as automotive, looking for biodegradable and renewable sources.

Beyond grain and seed by‐products, other wastes such as beer bagasse, rice and corn husks, are valuable resources for 3DP because they can contribute and enhance the mechanical and physical properties of the final product like tensile strength, flexibility, and thermal stability. For instance, 3D‐printed beer bagasse/PLA biocomposite filaments exhibit a tensile strength of 50 MPa and improved impact resistance by 35%, which are critical to ensure that packaging materials can withstand mechanical stress during handling and distribution (Carichino et al. 2023; Ohaeri and Cree 2022). Corn husk cellulose enhances the tensile strength of 3D‐printed PLA/cellulose composites to 42 MPa, providing the necessary flexibility and strength for biodegradable packaging and medical applications (Islam and Jiang 2022). Rice husk, containing 20% silica, improves stiffness and thermal stability up to 300°C, which makes the husk/silica composites suitable for construction materials even for applications that result in exposure to high heat, such as insulation and fire‐resistant building components (Morales et al. 2021). Wheat gluten is another valuable waste that helps structure strong 3D‐printed bioplastics with tensile strengths of 13.5 MPa, particularly useful in biodegradable packaging applications where durability and environmental friendliness are paramount (Alshehhi et al. 2024). Corncob powder and crude corncob extract, rich in lignocellulose, provide flexibility and easy processing for disposable consumer goods. The thermoreversible gel formed by crude corncob extract provides a tensile strength of 7.2 MPa, suitable for 3D‐printed biodegradable packaging and single‐use items (Gokce Bahcegul et al. 2022). All these examples highlight how grain and seed wastes can be repurposed to develop materials that meet both performance and sustainability goals across diverse industrial applications.

Nut processing wastes, including walnut shells, macadamia shells, and ground walnut shells, when mixed with copolyester (Co‐PES), exhibit a tensile, bending, and impact strengths of 2.0 MPa, 3.5 MPa, and 0.718 kJ/m^2^, respectively, making them ideal for lightweight, durable components for the automotive and construction sectors (Girdis et al. 2017; Y. Yu et al. 2017). Macadamia shells, when incorporated into acrylonitrile–butadiene–styrene (ABS) composites, reduced the density of the printed materials by up to 27.4%, while tensile strength improved by 25%, demonstrating their effectiveness in creating cost‐effective, lightweight structural components (Girdis et al. 2017).

Tree processing by‐products, like oil palm fibers, could improve some physical properties such as mechanical strength, flexibility, and thermal stability of 3D‐printed materials. Oil palm empty fruit bunch fibers, due to their high lignin and cellulose content, improved the tensile stress of ABS/graphene nanoplatelet composites to up to 29.1% with thermal stability reaching 280°C, compared with neat polymers making these composites highly suitable for electronics and automotive applications requiring both heat resistance and mechanical integrity (Mohan et al. 2021). These findings underline the versatility of tree waste in creating low‐cost, lightweight, and durable materials for demanding industrial environments.

#### 3.1.3 By‐Products From Fermentation and Extraction

Microbial fermentation and biorefinery approaches can add significant value to food by‐products in terms of nutritional, technological, and sensory characteristics (Ahmadzadeh et al. 2023; Jagadiswaran et al. 2021; Soh et al. 2023). Among these, spent coffee grounds (SCG) used as substrate to grow *Pleurotus ostreatus* mycelium and their consequent biocomposites (Soh et al. 2023), or green tea extract (GTE) and grape seed extract (GSE) (Ahmadzadeh et al. 2023) can confer important mechanical properties while also introducing important bioactive compounds in 3DP applications.

##### 3.1.3.1 Food‐Related Studies

Innovative 3D‐printed functional foods with high printability, mechanical stability, and fiber and antioxidant content have been obtained with 6% grape pomace inclusion into dough prepared using broken wheat (Jagadiswaran et al. 2021).

##### 3.1.3.2 Nonfood‐Related Studies

When combined with PLA, 20% SCGs enhanced the flexural modulus by 42%, making the PLA/SCG mixture ideal for applications requiring flexibility and structural durability, such as automotive interiors and flexible packaging (S. Li et al. 2021). Similarly, by adding 10% SCGs to low‐density polyethylene (LDPE), it was possible to significantly increase tensile strength by 20%; and by adding SCGs to high‐density polyethylene (HDPE) composites, thermal stability improved by 10°C–15°C, making these materials suitable for industrial packaging and heat‐resistant automotive components (Romani et al. 2024). Similarly, SCG increased the compressive strength of hydrogels by 25%, while also enhancing their thermal resistance, making them suitable for structural and architectural applications (Choi and Yi 2024). While the published documents on the use of SCG hydrogels had focused mostly on structural or architectural uses, no study has yet formulated a 3D‐printable food‐grade SCG gel. This would suggest that the development of edible systems based on SCG would also address the needs for fiber‐enriched functional gels or delivery systems for antioxidant or bioactive compounds in 3D‐printed foods. Overall, these results illustrate how these by‐products can be repurposed to create sustainable, high‐performance composites for a range of applications. Overall, these results illustrate how these by‐products can be repurposed to create sustainable, high‐performance composites for a range of applications.

#### 3.1.4 Biomass Processing By‐Product

Processing by‐products from biomass such as flax shives and *Miscanthus*‐derived biocarbon have demonstrated several benefits in 3D‐printable composite formulations. Badouard et al. (2019) reported that incorporating flax shives into a PLA–PBS blend at 10 wt% resulted in an increase in the Young's modulus of 3D‐printed specimens by approximately 6%, while maintaining acceptable surface quality and layer adhesion. In addition, the new material exhibited superior printability compared to fiber‐reinforced systems due to finer particle dispersion and reduced porosity in the printed layers. Likewise, Diederichs et al. (2021) showed that adding *Miscanthus* biocarbon to poly(trimethylene terephthalate) (PTT)–PLA filaments improved the thermal stability of printed parts, with 5 wt% biocarbon formulation reducing thermal expansion and improving dimensional precision without compromising tensile strength. These findings suggest that biomass by‐streams not only serve as sustainable fillers but also confer mechanical and thermal enhancements compatible with extrusion‐based 3DP (Figure 3).

### 3.2 3DP Techniques for Utilizing FBS Materials

**FIGURE 4:**
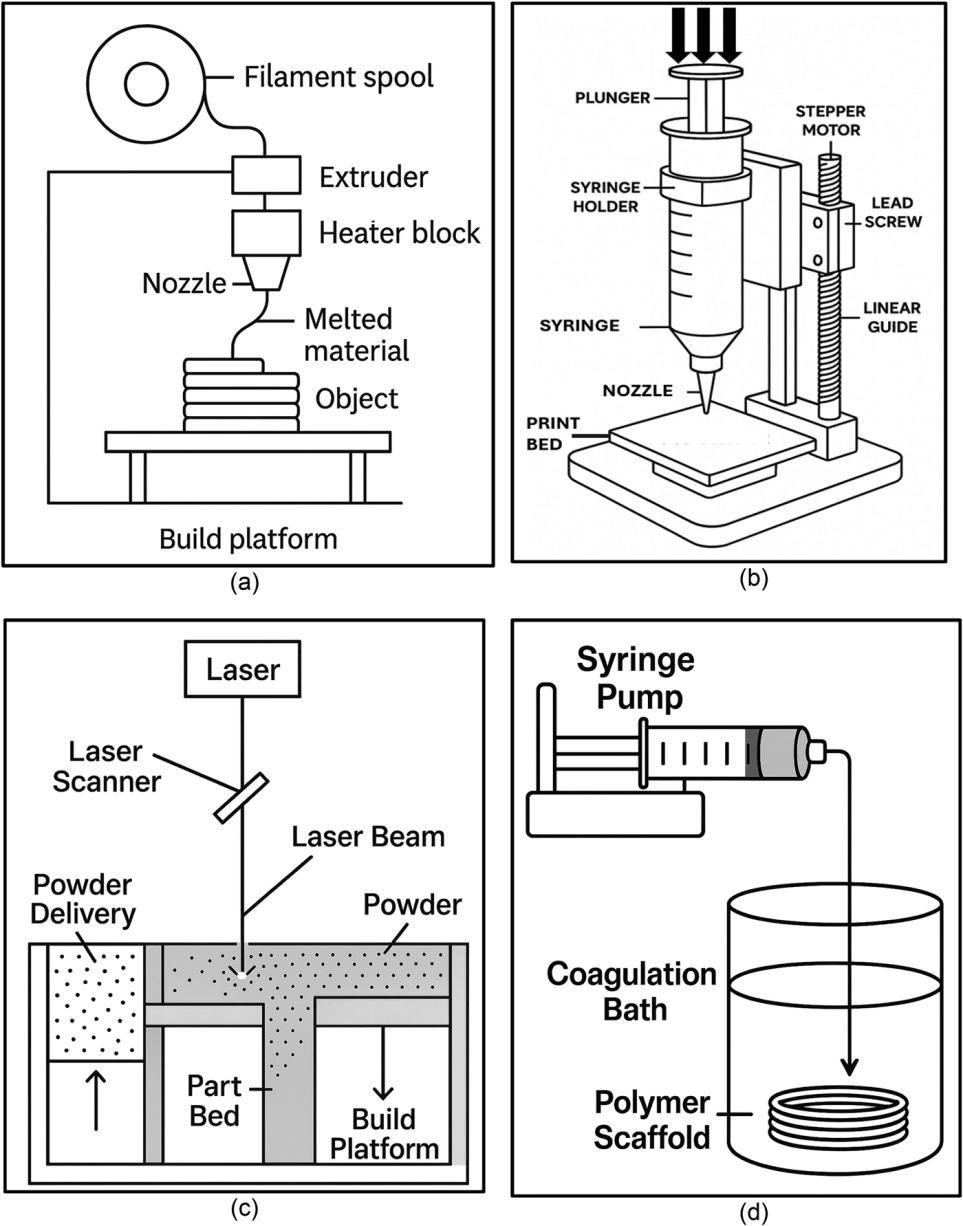
Schematic illustrations of commonly employed 3D printing techniques for utilizing food by‐stream materials. (a) Fused deposition modeling (FDM), (b) extrusion‐based 3D printing using a syringe‐based system, (c) selective laser sintering (SLS), and (d) computer‐aided wet‐spinning (CAWS) method.

A wide range of 3DP techniques can be employed to process various food by‐products aimed at creating thermoplastics, bioplastics, biomedical scaffolds, personalized foods, and building materials. The most commonly reported technology is material extrusion (ME), defined by ASTM as a process that involves the extrusion of a viscous or molten material through a nozzle (Derossi et al. 2024). This category encompasses methods such as fused deposition modeling (FDM), paste extrusion, and direct ink writing (DIW). Among the articles analyzed, 42% employed FDM as the primary 3DP method, while 31% utilized the paste extrusion technique, and DIW accounted for 18% of the studies. In contrast, only 7% of the surveyed articles focused on selective laser sintering (SLS). Furthermore, based on our results, only a limited number of experiments (<2) used computer‐aided wet‐spinning (CAWS), VAT photopolymerization, or stereolithography (SLA) as the main printing methods. Although a diverse set of 3DP techniques have been applied to FBS materials for upcycling, extrusion‐based technologies remain the most popular up to today. The following section provides an overview of such 3DP techniques (Figure 4), focusing on the printers employed and processing parameters, particularly printing variables utilized for effective fabrication of various types of materials.

#### 3.2.1 Fused Deposition Modeling

FDM processes which include fused deposition modeling, fused filament fabrication (FFF), and large‐format fused granular fabrication (FGF) were the preferred methods in the selected articles (42%). This preference stems from the most frequent application of food by‐products to reinforce and reduce the environmental impact of thermoplastics. The broad accessibility and versatility of FDM, ranging from low‐cost desktop printers to large‐scale industrial machines, as well as its feedstock requirements, that is, solid filaments, powders, or pellets, makes it an ideal platform for material innovation, particularly in the realm of sustainability and upcycling. FDM processing requires solid filaments with low viscosity upon heating and strong shear‐thinning behavior to flow smoothly through the nozzle (Figure 4a). Additionally, rapid solidification upon deposition is crucial to maintain dimensional accuracy and structural integrity. The incorporation of food by‐products into thermoplastic composites significantly enhances the performance and sustainability of 3DP materials. Cocoa bean shell (CBS) added to recycled polypropylene (rPP) composites, for example, reduced warping by 67%, improving dimensional stability during the printing process. This modification also increased the fracture strain by 118% in the 90°‐printed composite and enhanced water absorption by 138%, highlighting its potential to improve mechanical properties while reducing environmental impact (Morales et al. 2021b). ABS composites reinforced with Australian royal palm fiber offered notable advancements in sustainable 3DP applications. For instance, ABS composites with 15% palm fiber content showed improved and more extended fiber–matrix interactions, particularly to create biofilaments that can be used in 3DP pens. Moreover, the use of palm fiber in the composite slightly enhanced its thermal stability and led to smaller, more uniformly distributed voids providing structural benefits and less use of polymer resin, decreasing its environmental impact (e.g., ozone depletion (Marton et al. 2022)). Biocarbon derived from *Miscanthus* have been successfully integrated into PTT composites, intended for nonstructural automotive parts. The 5 wt% biocarbon composite exhibits impressive tensile strength (26.4 MPa) and a tensile modulus of 1.31 GPa, offering superior dimensional stability and surface finish, making it an effective solution for environmentally friendly applications. These examples underscore how food by‐products contribute to enhancing the mechanical and thermal properties of plastics, driving forward the sustainability and innovation of 3DP processes (Diederichs et al. 2021). A full summary of the advantages achieved in terms of mechanical and thermal properties by the addition of food by‐products is presented in Table 3.

**TABLE 3:** 3D printer details, printing parameters and effects caused by the incorporation of the food by‐stream materials in FDM applications.

Application	3D printer details	Material	3D printing conditions	Effect of the addition	Ref.
Biomedical applications	PRATHAM 3.0 FDM printer (custom made)	PLA and PLA/crab shell composite material	In: 100% PS: 20 mm/s Lt: 0.1 mm BT: 60°C	31% higher tensile strength than pure PLA Flexural strength improved by 47% Compressive strength reached 217 MPa. Overall impact strength about 1.3 kJ/m^2^	Palaniyappan et al. 2024
	FS‐200 FDM printer (Guangzhou Flythinking Intelligent Technology Co., Ltd., China)	PLA and PLA/crab shell composite materials	Nd: 0.4 mm In: 100% Nt: 200°C Bt: 50°C Lt: 0.1 mm Ps: 50 mm/s	Flexural strength about 90 MPa (28.8% improvement) Antibacterial activity was over 99% Excellent biocompatibility	Yang et al. (2023)
	Custom‐made FDM printer	PLA) and eggshell particle (ESP) composite	Nt: 210°C Bt: Not specified Nd: 0.4 mm Layer height: 0.1 mm In: 100% Ps: 60 mm/s	Composite scaffolds with 10 wt% ESP exhibited a maximum load‐bearing capacity of 369 MPa at 40% porosity Water contact angle decreased by 85% as ESP content increased, improving hydrophilicity and enhancing cell attachment The scaffold showed an 11 mg weight loss after 30 days in simulated body fluid, demonstrating significant degradation potential 12 wt% ESP scaffolds exhibited high cell viability (>95.2%), indicating excellent biocompatibility for bone tissue engineering	Gnanamani Sankaravel et al. (2022)
	Prusa i3 Hephestos, FDM printer (Prusa Research, Czech Republic)	Poly(ε‐caprolactone) (PCL) and micronized cocoa shell waste (CSW; 20%–50%) biofilaments	Nt:120°C Bt: Room temperature Nd: 0.6 mm Layer height: 300 µm (0.3 mm) In: 30% Ps: 50 mm/s	Biofilaments with 20 wt% CSW exhibited a Young's modulus, similar to pure PCL (334 vs. 304 MPa) Thermal stability sufficient for 3D printing Thermal instability around 387°C (50 wt% CSW) Printed objects with well‐connected layers and fine resolution (strong adhesion and uniformity)	Tran et al. (2017)
	Prusa i3 MK3S (Prusa Research, Czech Republic)	Acrylonitrile‐butadiene‐styrene (ABS) with graphene nanoplatelets (GnPs) as a reinforcer composite filaments	Nd: 0.6 mm Nt: 250°C Layer height: 0.30 mm In: 80% Ps: 60 mm/s Infill Pattern: 45° lines	ABS/5% lignin composite showed an increase in tensile stress by 16.2%, while addition of 5% lignin and 0.50% graphene nanoplatelets (GnPs) improved tensile stress by 29.2% compared to neat ABS The glass transition temperature (*T*_g_) of the ABS/5% lignin and 0.50% GnP composite increased to 105.8°C (*T*_g_ of neat ABS = 103.04°C)	Mohan et al. (2021)
	ENDER‐3S FDM printer (Creality 3D Technology Co. Ltd., China)	PLA and hydroxypropyl‐methylcellulose (HPMC) composite filaments	Nt: 200°C Bt: 40°C Nd: 0.4 mm Layer height: 0.2 mm In: 100% Ps: 50 mm/s	7% HPMC addition reduced tensile strength from 53 (pure PLA) to 49 MPa, due to increased porosity Cold crystallization temperature dropped from 111°C (neat PLA) to 104°C, indicating faster crystallization Enhanced hydrophilicity, with a 30° reduction in water contact angle, improving surface properties for biomedical applications	Jiang et al. (2020)
	Cuby DW‐300 FDM printer (Dimension Way Inc., Taiwan)	Filaments made from fish scale‐derived hydroxyapatite (FHAp), eggshell (EGS), and PLA	Nt: 190°C Bt: 55°C Layer height: 0.05 mm Ps: 150 mm/s (X‐axis) and 23 mm/s (Z‐axis) Filament diameter: 1.75 ± 0.05 mm	The PLA/6% FHAp composites exhibited a 7.9% increase in tensile strength compared to pure PLA, reaching 49.6 MPa Young's modulus also increased to 3.83 GPa Addition of 12 wt% EGS improved the antimicrobial properties, e.g., 64% inhibition zone against *Staphylococcus aureus*, compared to pure PLA Cell proliferation tests indicated improved cell attachment and growth, suggesting excellent cytocompatibility for potential use in bone regeneration and medical implants	Wu et al. (2021)
Environmental remediation and wastewater treatment	In‐house‐built FDM machine	TiO_2_/chitosan scaffolds	Syringe pump extrusion system equipped with a 0.192 mm diameter nozzle. Postprinting gelation by exposure to ammonia vapors	90% reduction in amoxicillin concentration in wastewater within 180 min under UV irradiation Photocatalytic efficiency remained high after three cycles (80% degradation in the final cycle)	Bergamonti et al. (2019)
	Sharebot Next Generation FDM printer (Sharebot, Italy)	Biodegradable green composites of PLA and tomato plant waste	Nt: 160°C (for MB) and 190°C (for PLA) Bt: 60°C (MB) and 90°C (PLA) Nd: 0.4 mm Lt: 0.1 mm In: 100% Raster angle: ±45° Ps: 50 mm/s	100% release of NPK fertilizer after 30 days Removal efficiency of Cu(II) ions = 78%	Scaffaro et al. (2024)
	Sharebot Next Generation FDM printer (Sharebot, Italy)	Biodegradable green composites from Mater‐Bi (MB) and tomato plant waste	Nt: 160°C Bt: 60°C Nd: 0.4 mm Lt: 0.1 mm In: 100% Raster angle: 0° or ±45° Ps: 50 mm/s Extrusion width: 0.4 mm	Tensile strength of 19.1 MPa (5% tomato plant waste) vs. 17.5 MPa (10% tomato plant waste) Elastic modulus of 540 MPa (5% inclusion), copper ion adsorption of 78% (10% composite) Filler addition improved biodegradability and environmental impact reduction potential	Scaffaro et al. (2023)
Drug delivery	UP Mini 3D printer (3D Printing Systems, New Zealand)	Negative molds using acrylonitrile‐butadiene‐styrene (ABS) filaments	Indirect 3D printing used just to create molds rather than directly printing the gelatin products	High crosslinking extension Mass loss of only 8.5% after 7 days in a fatty food simulant Fast initial release of gallic acid (34% in 8 h) followed by a sustained release over 10 days	Stevenson et al. (2019)
	Ultimaker 2+ Connect FDM printer (Ultimaker B.V., The Netherlands)	PLA blended with food wastes (e.g., brewer's spent grain (BSG), spent coffee grounds (SCG), and sesame cake (SC))	Nd: 0.4 mm Nozzle temperature: 178°C (PLA‐BSG, PLA‐SCG, PLA‐SC) Bt: 70°C Ps: 30 mm/s In: 100% Layer height: 0.27 and 0.60 mm	Up to 15% food waste could be incorporated into PLA filaments Drug release studies revealed a burst release of metoprolol tartrate (MT) from the composites, with complete release within 2 h for all samples Wall thickness of the printed capsule significantly affected drug release (i.e., thicker walls = slower release rates) SEM images showed that food by‐products particles enhanced the porosity of the capsule, accelerating drug diffusion	Wang et al. (2024)
Packaging and sustainable material development	Original Prusa i3 MK3S+ (fused filament fabrication (FFF) printer; Prusa Research, Czech Republic)	3D‐printable polymer composite from PLA and tomato stem powder (5 & 10%)	Nt: 215°C Bt: 60°C Nd: 0.8 mm Layer height: 0.3 mm In: 100%	Tensile stress of 44.3 MPa (5% tomato stem) vs. 32.4 MPa (10% tomato stem) Young's modulus of 1719 MPa High antioxidant activity due to tomato stem addition (10% composite 30% less residual DPPH content than 5%	Pemas et al. (2024)
	FF‐STD Doppia fused filament fabrication (FFF) printer (Simplify 3D, Cincinnati, USA)	Recycled polypropylene (rPP) and cocoa bean shell (CBS; 5%) composite filaments	Nt: 250°C Bt: 90°C for the first layer, 70°C for subsequent layers Nd: 0.8 mm Layer height: 0.25 mm Ps: 60 mm/s In: 100% Raster Angles: 0° and 90°	Reduced warping effect by 67%, improving dimensional stability during 3DP Fracture strain increased by 118% in the 90°‐printed composite vs. neat rPP Water absorption increased by 138% with the introduction of CBS	Morales, et al. (2021b)
	Tumaker NX Pro Pellets (Tumaker, Spain) for pellets and BQ Witbox 2 (BQ, Spain) for filaments	PLA and beer bagasse biocomposites (pellets and filaments)	Nd: 0.8 mm (both printers) Lt: 0.4 mm Extrusion multiplier: 1.1 (Witbox) & 7.5 (Tumaker) Ps: 40 mm/s (Witbox), 30 mm/s (Tumaker) In: 100% Nt: 190°C (both printers) Bt: 50°C (Witbox), 60°C (Tumaker)	The addition of beer bagasse (10 wt%) increased the Young's modulus of the composite (613 (composite) vs. 502 MPa pure PLA) Addition of epoxidized linseed oil (ELO) as compatibility improved the impact strength to 19 kJ/m^2^ (vs. 35 kJ/m^2^ for pure PLA) Tumaker NX Pro Pellets showed superior dimensional accuracy and fewer voids compared to those printed with BQ Witbox 2	Carichino et al. (2023)
	Ultimaker3 FFF 3D printer (Ultimaker, the Netherlands)	PHB/PLA and corncob composite filaments	Nt: 200°C Bt: 50°C Nd: 0.6 mm Layer height: 0.1 mm Ps: 50 mm/s In: 100% Infill pattern: Concentric Fan speed: 100%	PHB/PLA with 6 wt% corncob filler exhibited a 6% increase in tensile modulus, while its tensile strength slightly decreased 8 wt% corncob composite showed the highest water absorption (300% higher than pure PHB/PLA) Impact strength decreased by 6%–8% as filler loading increased	Ohaeri and Cree (2022)
	Ultimaker 3 Extended FDM printer (Ultimaker B.V., the Netherlands)	Poly(butylene adipate‐co‐terephthalate) (PBAT) composite reinforced with flax shives	Nt: 190°C Bt: 55°C Nd: 0.8 mm Layer height: 0.2 mm In: 100% with octahedral pattern	Addition of 10 wt% flax shives resulted in a 17.8% decrease in Young's modulus and a 5.2% reduction in strength compared to injected samples Fluorescence intensity remained strong, despite successive heating steps, indicating the potential for sensor applications with complex 3D‐printed shapes	Mayer‐Laigle et al. (2021)
	CR200B FDM printer (Creality 3D Technology Co. Ltd, China)	Spent coffee grounds (SCG) and spent tea leaves (STL) composite filaments with PLA	Nt: 200°C Bt: 50°C Nd: 0.4 mm Layer height: 0.2 mm In: 100% Ps: 30 mm/s	PLA/20% SCG composites had lower tensile strength than pure PLA (18.2 vs. 50.2 MPa) Elongation at break increased fivefold to 33.4% due to the plasticizing effect of the SCG oil Thermal degradation analysis showed minimal mass loss below 185°C, ensuring stability during 3D printing	Yu and Wong (2023)
	Prusa i3 Rework FDM printer (Prusa Research, Czech Republic)	Fully compostable composite PLA and flax fibers or shives composites	Nt: 190°C (PLLA) and 150°C (PBAT) Bt: 70°C Nd: 1 mm Layer height: 0.6–1.0 mm In: 100% Ps: 0.8–1.5 mm/min	10% Flax‐reinforced PBAT composites showed excellent flexibility, with 364% elongation at break Higher fiber content (30%) in PBAT led to extrusion instability, impacting printability but still improving the mechanical properties compared to the base polymers	Badouard et al. (2019)
	Sharebot Next Generation FDM printer (Sharebot, Italy)	PLA and anchovy fishbone powder filaments	Nt: 160°C (for Mater‐Bi) and 240°C (for PLA) Bt: 60°C Lt: 0.1 mm Extrusion width: 0.4 mm In: 100% Ps: 50 mm/s	Improved elongation at break (3.8% vs. 5.6%) Flexural modulus increase of 32%	Scaffaro et al. (2022)
Automotive and industrial components	LulzBot Taz 6 FFF printer (Aleph Objects, Inc., USA)	Poly(trimethylene terephthalate) (PTT) and biocarbon (BC) composites made from *Miscanthus*	Nt: 280°C Bt: 65°C Nd: 0.5 mm Layer Height: 0.3 mm Ps: 35 mm/s	PTT/5% BC composites exhibited comparable mechanical performance to PTT/10% BC composites but provided superior dimensional stability and surface finish The tensile strength of the 5 wt% BC composite was 26.4 MPa, while the tensile modulus was 1.31 GPa, making it suitable for nonload‐bearing applications in the automotive industry	Diederichs et al. (2021)
	3D FF‐STD Doppia FDM printer (Simplify 3D, USA).	Recycled polypropylene (rPP) and rice husk (RH) composite filaments	Nt: 240°C Bt: 90°C for the first layer, 70°C for subsequent layers Nd: 0.8 mm Layer height: 0.25 mm In: 100% Ps: 60 mm/s Raster angles: 0° and 90°	rPP/RH composites (10 wt% RH) exhibited 13.8 MPa tensile strength when printed at a 0° raster angle and a slight improvement in Young's modulus compared to pure rPP RH addition improved dimensional stability, reducing the warping during printing Water absorption increased significantly due to the hydrophilicity of RH, e.g., rPP/RH composite absorbed 300% more water than rPP alone	Morales, et al. (2021a)
	Leapfrog Creatr FDM printer (Leapfrog, the Netherlands)	Macadamia nutshell (19%–29%) and acrylonitrile butadiene styrene (ABS) composite filaments	Nd: 0.5 mm In: 30% (for compression samples), 100% (for tensile samples) Filament diameter: 1.75 mm	Macadamia nutshell/ABS filaments exhibited a 25% increase in specific tensile strength than commercial wood polymer composites The composite displayed a 60% increase in ductility compared to traditional wood‐based composites Addition of 29% macadamia nutshells reduced the composites' density by 27.4%, making it significantly lighter than pure ABS	Girdis et al. (2017)
	Weellzoom B Desktop FDM printer, (Weellzoom, China)	Acrylonitrile‐butadiene‐styrene (ABS) and Australian royal palm fiber composite filaments	Fiber Loadings: 5%, 10%, 15%, and 20% by weight of palm fibers Filament diameter: 1.75 mm Porosity: Fiber loading affected porosity, with smaller pores (< 50 µm) at higher fiber content (15%–20% palm fiber)	ABS/palm fiber composites (15%) had a 42% higher hydrogen‐bonding coefficient than pure ABS, indicating better fiber–matrix interaction The heat resistance index of ABS/palm fiber composites showed minimal changes, with a slight improvement at 15% fiber content due to hydrogen bonding The Life Cycle Assessment (LCA) indicated environmental benefits including lower agricultural land use and ozone depletion More even and better distributed void content in the 15% fiber composite than in lower fiber content composites	Marton et al. (2022)
	Prusa i3 MK3 FDM printer (Prusa Research, Czech Republic)	Poly(lactic acid) (PLA) and buckwheat husk (BH) composites	Nd: 0.8 mm Layer height: 0.15 mm Nt: 215°C Bt: 60°C In: 100% Ps: 50 mm/s for perimeters, 80 mm/s for solid infill	Adding 10% BH filler increased PLA's crystallinity from 7 to 19% Tensile strength decreased from 52.2 MPa (pure PLA) to 38 MPa (BH addition) The elongation at break for PLA/BH composites was recorded at 3.4%, which is higher than the pure PLA (2.6%), showing good ductility with the filler	Andrzejewski et al. (2021)
	Lulzbot Taz 6 FFF printer (Lulzbot Taz 6, USA).	Recycled high‐density polyethylene (rHDPE) and recycled polypropylene (rPP) composites, reinforced with pyrolyzed soy hulls biocarbon	Nd: 0.5 mm Layer height: 0.3 mm Line width: 0.7 mm Ps: 35 mm/s In: 100% Nt: 200°C–235°C Bt: 80°C–100°C Line pattern: −45°/45°, 90°, 180°	rHDPE/rPP reinforced with 20% biocarbon's Young's modulus and flexural modulus increased by 15% and 11%, respectively, than rHDPE/rPP without biocarbon Warpage and dimensional stability improved by increasing bed and nozzle temperatures Lower warpage angle when biocarbon and malleated polymers were used as compatibilizers	Maldonado‐García et al. (2021)
	FELIX Pro 1 FDM printer (FELIXprinters, the Netherlands).	PLA and micro/nano‐structured white spent coffee grounds (MN–DSCGs) composites	Nt: 195°C Bt: 40°C Nd: 0.5 mm Layer height: 0.2 mm In: 100% Ps: 30 mm/s	PLA/ MN‐DSCGs blended matrices had a comparable mechanical performance, i.e., similar tensile strength, to pure PLA Flexural modulus increased by 42% in samples with 20% MN–DSCGs	Li et al. (2021)
	RAISE3D E2 printer (Raise3D, I, USA)	Polyhydroxybutyrate (PHB) and poly(methyl methacrylate) (PMMA) blend filaments, chemically crosslinked with dicumyl peroxide (DCP)	Nd: 0.4 mm Nozzle temperature: 200°C Bt: 55°C Ps: 25 mm/s In: 100% Layer height: 0.2 mm Filament diameter: 1.75 mm	PHB/PMMA blends (1:2 ratio) with 0.3 wt% DCP showed higher tensile strength than PHB and PMMA (78.6 vs. 30.2 vs. 59.4 MPa) Elongation at break increased by 10.9%, showing enhanced ductility compared to neat PHB showing enhanced ductility Biodegradation studies revealed 41.3% efficacy in soil after 60 days and 40.4% efficacy during enzymatic degradation after 50 days	Rofeal et al. (2023)
Large‐format 3D printing	Delta Wasp 3MT large‐format fused granular fabrication (FGF) printer (Wasp S.R.L., Italy)	PLA and spent coffee grounds (SCG) composite pellets.	Nt: 190°C Bt: 80°C Nd: 3 mm Layer height: 0.5–1.2 mm In: 100% for characterization samples Ps: 10–30 mm/s	PLA/10% SCG composites displayed a 10% lower elastic modulus than neat PLA, while maintaining a tensile strength of 46.8 MPa The use of SCGs reduced viscosity, improving printability and layer adhesion, and facilitating 3D printing of large‐scale products, e.g., coffee table, with no visible defects or clogging during extrusion	Paramatti et al. (2024)
	Delta Wasp 3MT large‐format fused granular fabrication (FGF) printer (Wasp S.R.L., Italy)	PLA and spent coffee grounds (SCG) composites	Nt: 190°C (PLA/SCGs), 170°C (LDPE/SCGs and HDPE/SCGs) Bt: 80°C (PLA/SCGs and LDPE/SCGs), 60°C (HDPE/SCGs) Nd: 3 mm Layer height: 0.5, 0.7, and 1 mm (depending on the material) In: 100% for tensile samples, 0% (no infill) for 3D‐printed vase mode samples Ps: 10 mm/s (feed rate)	PLA/SCGs composites exhibited an elastic modulus of 1547 MPa and a tensile strength of 13.9 MPa LDPE/SCGs composites had an elastic modulus of 107.4 MPa and a tensile strength of 6.5 MPa HDPE/SCGs composites showed a tensile strength of 10.9 MPa and an elastic modulus of 587 MPa Nonplanar slicing allowed for the successful fabrication of complex overhangs up to 32° curvature without support, showcasing the potential for large‐format applications	Romani et al. (2024)

Abbreviations: Bt, bed temperature; In, infill percentage; Nd, nozzle diameter; Nt, nozzle temperature; Ps, printing speed.

To successfully develop FDM‐suitable materials, key process parameters such as extrusion speed, nozzle and bed temperatures, filament flowability, and nozzle diameter must be optimized to ensure desired properties and functionalities. Based on the analyzed studies herein, the extrusion speeds varied significantly from 20 to 50 mm/s, depending on the materials and intended applications. 3DP of PLA/crab shell polymeric composites utilized speeds from 20 to 50 mm/s, with 40 mm/s attaining optimal printing quality and mechanical properties (Palaniyappan et al. 2024). In addition, hydroxypropyl‐methylcellulose‐reinforced PLA (HPMC/PLA) biocomposite filaments with 100% infill were printed at optimal speed of 50 mm/s (Jiang et al. 2020). Overall, while faster extrusion speeds may increase production efficiency, they sometimes compromise filament uniformity, whereas slower speeds often enhance precision at the cost of productivity.

The temperature at the nozzle and on the bed surface plays a pivotal role in ensuring proper layer adhesion and preventing warping. The literature analyzed generally reported nozzle temperatures from 120°C to 280°C, while bed temperatures varied from room temperature to 100°C. For instance, a study using cocoa shell waste‐incorporated PCL bioplastics required a relatively low nozzle temperature, 120°C, due to the thermal sensitivity of the PCL (Tran et al. 2017). In contrast, studies that involved composite filaments made of fish scale‐derived ethylenediaminetetraacetic acid‐functionalized fluorapatite (EFHAp)–PLA blends required higher nozzle temperatures, that is, between 190°C and 200°C to ensure proper melting and extrusion (Wu et al. 2021). The highest nozzle temperature reported was 280°C in studies incorporating *Miscanthus* biocarbon into Sonora PTT thermoplastic composites, due to the high thermal energy needed to process rigid biocarbon fillers (Diederichs et al. 2021). Another important printing variable is nozzle diameter, which exhibits values between 0.25 and 3 mm, based on the material properties and printing objectives. The thinnest nozzle (0.25 mm) was used for printing fine‐detail gastric floating drug delivery systems made of PLA blended with agro‐industrial by‐products (L. Wang et al. 2024). The most frequently used nozzle diameter was 0.4 mm, which ensured an adequate balance between precision and extrusion efficiency; this condition was applied in multiple studies involving PLA‐based composites, including PLA/crab shell composites and other sustainable polymer composite material development studies (Jiang et al. 2020; Palaniyappan et al. 2024; Scaffaro et al. 2024; Wu et al. 2021). On the other hand, larger nozzle diameters, such as 0.5–0.6 mm, were employed in more complex formulations like ABS/palm fiber composites and biocarbon‐enhanced composites, where structural integrity was critical (Diederichs et al. 2021; Marton et al. 2022). For large‐scale applications, such as development of PLA/corncob composites and fluorescent biocomposites, a 0.8‐mm nozzle was used to facilitate faster printing and better material flow (Mayer‐Laigle et al. 2021; Ohaeri and Cree 2022). The largest nozzle diameter utilized in the studied literature, 3 mm, was used in large‐format FGF studies, where high structural strength and large build volumes were essential (Paramatti et al. 2024). To better exemplify the versatility of FDM as the most frequently employed 3DP technique for upcycling food by‐products into various functional materials, Table 3 provides a thorough analysis of the pertinent studies. This table includes detailed information on the 3D printers used, key printing parameters, and significant findings observed across the studies.

#### 3.2.2 Paste Extrusion

Paste extrusion, encompassing both semi‐solid extrusion (SSE) and hot melt extrusion (HME), has been widely utilized for the upcycling of FBS materials. The analysis of the literature found 22 articles describing sustainable solutions in food manufacturing, biodegradable packaging, and biomedical applications. SSE involves the extrusion of semi‐solid materials such as pastes, gels, or other high‐viscosity substances, typically using mechanical force applied via a piston, screw, or pneumatic pressure (Figure 4b). Overall 3DP conditions for SSE consist of ambient‐to‐moderate temperatures (≈ 20°C–40°C) under pneumatic or screw pressures of 0.05–0.60 MPa, whereas HME operated at melt temperatures of 100°C–140°C (typically 120°C for PCL‐based scaffolds) with the melt conveyed under comparable mechanical pressures. A total of 12 published manuscripts have focused on 3DFP. The remaining 10 studies provide information on the use of paste extrusion in biodegradable packaging, sustainable construction, and biomedical fields which are the focus of this section. In the realm of sustainable construction, a novel approach was presented by combining seashell powder from *Aequipecten* species with *Pleurotus ostreatus* mycelium for biowelding in 3D‐printed biodigital bricks (Abdallah and Estévez 2023). The powder consisting of 95%–97% CaCO_3_ with minor mineral and organic fractions provide a calcium‐rich substrate that stimulates mycelial growth. As the brick cures, chitinous hyphae infiltrate successive 1 mm layers and bioweld them, creating a carbon–calcium network that binds the CaCO_3_ platelets into a coherent, mortar‐free structure. Using paste extrusion, each brick was composed of five layers, each 1 mm thick, and a full V3 linear brick model, consisting of 34 layers, was found to be 40% lighter than a similarly scaled V3‐LBM printed in clay (1000 vs. 1658.66 g). The seashell content of the bricks was approximately 80% after drying, and SEM and EDX analyses confirmed effective biowelding. Another study developed a biocomposite ink for 3DP using xanthan gum, chlorella, cellulose, eggshell, and charcoal (Choi and Yi 2024). The final ink formulation exhibited compressive strengths from 6.50 to 9.67 MPa, with an average of 8.69 MPa, and flexural strength averaging 8.78 MPa, demonstrating sufficient structural stability for large‐scale architectural printing. Additionally, Bhardwaj et al. (2021) examined the impact of psyllium husk powder on the print quality of biomass–fungi composite materials. Psyllium husk powder water ratios (w:v) of 1:40 and 2:40 demonstrated good extrudability and dimensional consistency. In the composite, increasing the husk‐to‐water ratio from 0:40 to 2:40 (w/v) raised the loss tangent (tan *δ*) from 0.19 to 0.28, converting a nonextrudable paste into one that printed continuous filaments 8.68 ± 0.22 mm wide with only 2.5% width variation and just 5% height loss after deposition of a second layer, parameters that met the authors’ ±5% shape‐stability criterion. Raising the ratio further to 3:40 resulted in a reduction of tan *δ* to 0.23, causing filament tearing and confirming that an intermediate tan *δ* (∼0.28) gives the optimal balance between flow and structural retention. SSE was also applied to create sustainable packaging derived from food by‐products such as banana peel powder combined with guar gum (Nida et al. 2022, 2023). Two companion studies on banana‐derived residues show how adding guar‐gum (GG) improved rheological properties and, in turn, printing quality. For banana‐peel (BP) powder, 1% w/w GG raised the consistency index (k) from 2.17 × 10^3^ to 2.29 × 10^3^ Pa sⁿ while retaining shear‐thinning behaviour (*n* = 0.28); apparent viscosity was ≈ 10 Pa s at a shear rate of 60 s^−1^. This flow window allowed smooth extrusion at 600 kPa through a 1.2‐mm nozzle, with optimum conditions of 150 rpm screw speed, 500 mm/min travel speed, a printing rate of 0.186 ± 0.002 g/min and a critical nozzle height of 0.8 mm, giving layer‐stable casings free of seams or sagging (Nida et al. 2023). When BP was blended with SCB, GG once again enabled extrusion: BP:SCB inks in 1:1 and 9:1 ratios printed cleanly at 3.2 bar and 1.2‐mm nozzle diameter; the 1:1 blend required 400 mm/min and a critical nozzle height of 1.34 mm, whereas the 9:1 blend performed best at 600 mm/min and a critical nozzle height of 0.54 mm, adjustments that eliminated under‐ or over‐extrusion gaps. Across both formulations, GG addition increased cohesiveness and springiness while reducing hardness, producing self‐supporting walls with ≤ ±3% dimensional deviation after drying (Nida et al. 2022). In another study, the properties of hybrid printed bioplastic films combining plant‐derived gluten and animal‐derived keratin proteins were described (Alshehhi et al. 2024). These films, fabricated using a ruthenium‐based photocrosslinking method, exhibited enhanced water uptake, increased molecular chain mobility, and tunable biodegradability, making the complex ink suitable for 3DP in packaging and biomedical applications. SSE was employed to develop oral films for personalized pharmaceuticals using carboxymethyl cellulose (CMC) derived from durian rind waste (Panraksa et al. 2023). This approach allowed obtaining programmable drug release profiles by modifying printing parameters such as infill density and pattern design. For instance, grid‐patterned films exhibited greater porosity, resulting in faster drug release. Soy protein hydrogels enhanced with cellulose‐containing residue from agar production were used for tissue engineering scaffolds (Uranga et al. 2023), showing a suitable viscosity, that is, 12 Pa·s at a shear rate of 90 s^−1^, for extrusion through a 1.6‐mm nozzle, maintaining shear‐thinning behavior for effective flow and shape retention postdeposition. Similarly, HME was used to produce PCL/nano‐hydroxyapatite (HA) scaffolds for bone tissue engineering (Cestari et al. 2021). HA synthesized from cuttlefish bones, mussel shells, and eggshells was blended with PCL to create scaffolds mimicking trabecular bone structures. The scaffolds showed improved mechanical properties, such as increased elastic modulus and yield stress, and enhanced cell adhesion, proliferation, and metabolic activity, particularly in those containing mussel shell–derived HA. These studies collectively demonstrate the versatility of paste extrusion techniques in promoting sustainability and functionality across various sectors. By upcycling food by‐products into high‐performance materials for construction, packaging, and biomedical applications, these innovations provide eco‐friendly solutions capable of being implemented on a large scale.

#### 3.2.3 Direct Ink Writing

DIW utilizes extrusion‐based deposition but involves more fluid materials, such as gels or slurries, that require postprocessing, like curing or drying, to ensure structural stability. Various food by‐products, such as orange peels (Da Tan et al. 2023; Leo et al. 2022) durian husks (Tan et al. 2022), okara (a by‐product of soybean processing) (Lee et al. 2021), PPP from slaughterhouse waste (Álvarez‐Castillo et al. 2021), and SSG (Carvajal‐Mena et al. 2022), have been studied as materials to improve the functionality of 3D‐printed foods and other nonedible materials. For instance, specialized inks have been developed for electromagnetic interference (EMI) shielding (Sanandiya et al. 2024), thermally tunable biocomposite inks (Islam and Jiang 2022), and biomass‐derived bioplastics for multiproduct biorefineries (Gokce Bahcegul et al. 2022). Innovative applications include using nonfluid agar gel–based inks for 3D‐printed mycelium structures (Soh et al. 2023) as potential self‐healing materials. Durian husk‐based inks showed yield stresses from 672 to 9755 Pa, depending on the concentration of rheology modifiers (Tan et al. 2022). It should be noted that yield stresses between 500 and 1500 Pa are recommended for good extrudability and shape retention. Chitosan‐based electroconductive inks were used for EMI shielding and strain‐sensing applications. In this case, ink formulation with a loading of 66.7% carbon fibers (CF) improved electrical conductivity, tensile strength (20.8 MPa), and Young's modulus (3.92 GPa), demonstrating that the material could maintain structural integrity while also serving as an effective conductor (Sanandiya et al. 2024). Another article described the effect of adding crude corn cob extracts rich in hemicellulose and lignin on the mechanical properties of nonfood materials. The printed self‐supporting structures, with a tensile strength of 7.2 MPa and a Young's modulus of 653.7 MPa, were stable under various conditions (Gokce Bahcegul et al. 2022). Corn‐based thermally tunable biocomposites were developed as sustainable materials with enhanced mechanical properties and printability. By combining cellulose fibers from corn husks with a corn starch matrix, the composites exhibited a 3.3‐fold increase in mechanical strength after thermal treatment, with a storage modulus reaching 648 MPa (Islam and Jiang 2022). Additionally, print speeds of 10–20 mm/s and layer heights of 0.4 mm contributed to the structural integrity and smooth surface finish of the printed parts.

#### 3.2.4 Selective Laser Sintering

SLS applications constituted a smaller proportion (7%) of selected research articles. However, this technique shows great potential for creating 3D‐printed structures incorporating food by‐products. SLS uses a high‐power laser to sinter‐powdered materials, binding them together to form solid structures. In SLS, the unsintered powder acts as a natural support for the part during printing, enabling the creation of complex geometries by precisely directing the laser to sinter only the designated areas of the powder bed (Figure 4c). Laser power, scan speed, and layer thickness are critical factors that influence the sintering process. Previous studies have investigated the use of various biomass derivatives, such as walnut shell powder, rice husks, bamboo, and pine wood, as feedstocks to produce sustainable and high‐performance biocomposites using SLS. Walnut shell powder was extensively studied due to its ease of preparation, affordability, and beneficial properties for SLS. Y. Yu et al. (2021) mixed walnut shell powder with copolyester (Co‐PES) to develop a low‐cost, eco‐friendly material for SLS. Walnut shell powder particle size between 80 and 120 µm provided the best mechanical properties, with a tensile strength of 2.01 MPa, bending strength of 3.5 MPa, and impact strength of 0.718 KJ/m^2^_._ This study showed that smaller particle sizes led to higher surface roughness, while larger sizes affected the internal density and porosity of the final product (Y. Yu et al. 2017, 2021). Smaller particle sizes (≤ 58 µm) led to particle agglomeration and a lack of sufficient material to fill voids during the sintering process, which resulted in rough surfaces. Conversely, particle sizes within the medium range (e.g., 80–120 µm) generate smoother surfaces due to more effective packing while larger particles that cannot fit as effective together also result in formation of voids and higher internal porosity, not necessarily increasing surface roughness but reducing density and mechanical integrity. Larger particles can also be unevenly distributed on the surface, worsening the roughness when the size difference is too large. One study investigates rice husk (RH)/Co‐polyamide (Co‐PA) composites for SLS applications. The study identified that a 10 wt% of RH addition provided the best balance between mechanical properties and dimensional accuracy (Li et al. 2023b). The SLS process parameters were optimized, using a laser power of 16 W, scan speed of 2 mm/s, layer thickness of 0.25 mm, and a preheating temperature of 74°C, resulting in a final product with high accuracy and suitability for investment casting. In another study, PBAT‐based composites were filled with agro‐wastes, such as corn germ meal and wine production by‐products. These fully biodegradable composites were used for the first time in SLS, with the resulting materials exhibiting enhanced mechanical properties and porosity suitable for biomedical applications. The addition of 5–10 wt% biofillers into the PBAT matrix allowed sustainable 3DP, with optimized SLS parameters such as a layer thickness of 0.1 mm and a laser scan speed of 2400 mm/s. These composites were characterized by high dimensional accuracy and excellent biodegradability, making them ideal for fields such as biomedical devices and food packaging (Colucci et al. 2024).

#### 3.2.5 Miscellaneous Methods

Several less conventional 3DP methods have also been explored in the reviewed studies. One such technique, CAWS method, involves the extrusion of a hydrogel‐forming polymer mixture into a nonsolvent bath to create 3D scaffolds (Figure 4d). This method, particularly suited for biomedical applications, requires precise control of the overflow rate and needle movement. A flow rate of 0.2 mL/h and a translational velocity of 540 mm/min were identified as the optimal condition for maintaining structural integrity during scaffold fabrication (Braccini et al. 2024). Fine control over layer thickness, such as a 50 µm Z‐axis translation, ensured uniformity and functionality of the scaffold structure. VAT photopolymerization was employed using an ASIGA UV‐MAX X27 DLP printer, which leverages photocurable resins to produce biodegradable composites. The resin, comprising 5%–33% microcrystalline cellulose (MCC) from aloe vera waste showed optimal printability at 20% MCC, achieving high resolution and accuracy with a 27 µm XY resolution and minimal deviation from CAD models (Cabua et al. 2024). A similar approach in SLA utilized a 405‐nm UV laser with a 0.2‐mm layer height, achieving a good balance between surface smoothness and mechanical strength (Romero‐Ocaña et al. 2022).

### 3.3 Applications for FBS Materials in 3DFP

Various studies proved the potential of FBS material as functional ingredients in 3DFP, contributing to the development of high‐quality, sustainable, and tailored food products. This section delves into the diverse applications of FBS materials in improving the printability of food inks, enhancing their nutritional profile, and optimizing sensory properties such as taste, color and texture.

#### 3.3.1 Use of FBS Materials to Improve the Printability of Food Inks

##### 3.3.1.1 Effect of Fiber‐Rich FBS Materials

**FIGURE 5:**
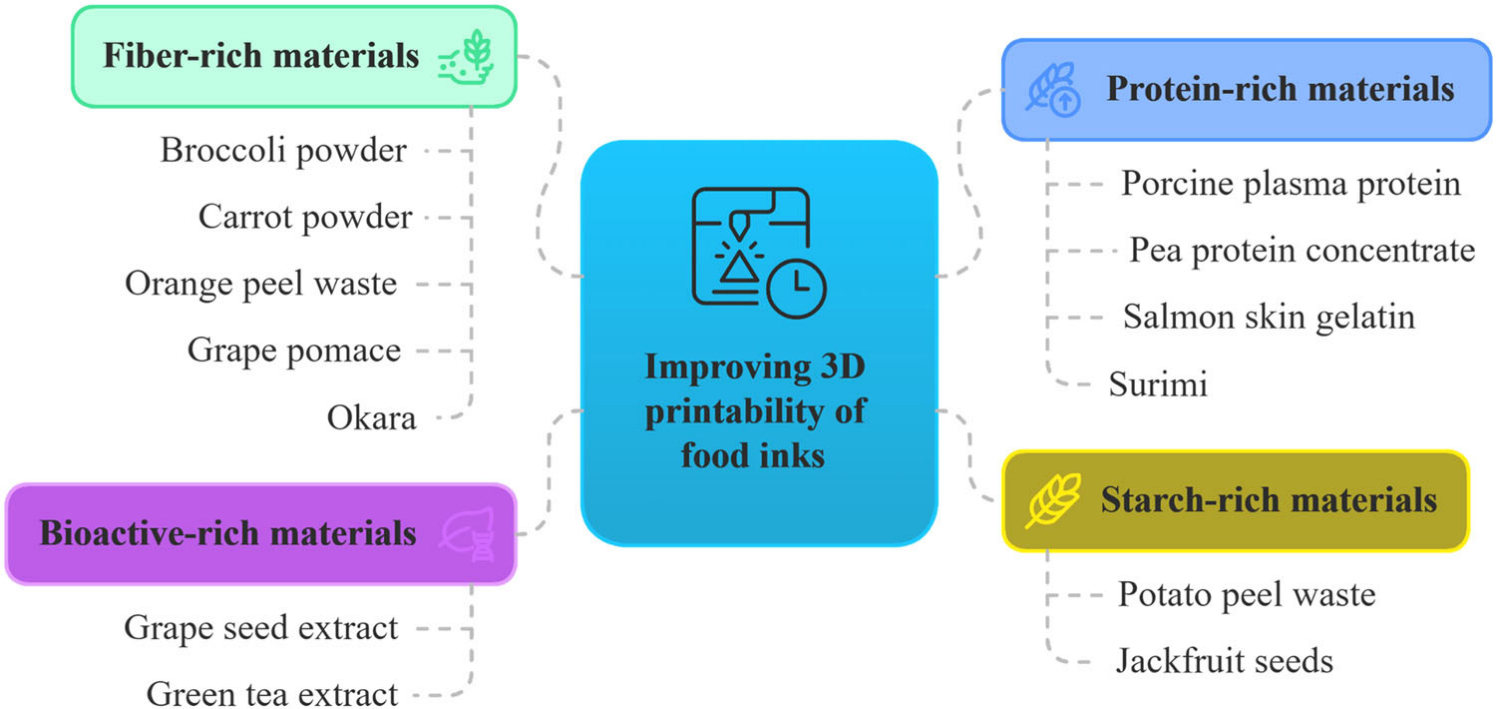
Categories of food by‐stream materials for enhancing food ink printability: Fiber‐rich, protein‐rich, bioactive‐rich, and starch‐rich materials. These materials contribute distinct functional properties, such as improved consistency, gelation, and viscosity, facilitating effective 3D printing of food inks.

Incorporating FBS materials into 3D‐printed snack formulations significantly enhances their printability by modifying the rheological properties of the dough or paste (Figure 5). By leveraging the high fiber content, water retention capabilities, and structural benefits of various food by‐products, researchers have successfully developed high quality 3D‐printed food. For example, the incorporation of freeze‐dried broccoli and carrot powders into wheat flour‐based doughs introduced significant modifications into the dough matrix, by modifying its water absorption properties thus altering the viscosity of the food formula. Broccoli powder additions at a 50% level generated a notable increase in viscosity of 10,000 Pa·s compared to 1000 Pa·s in control sample, allowing for better control of flow and improved shape retention. In addition, given the broccoli's ability to swell up to 7.6 times its original size when hydrated, a strong internal network within the dough was observed resulting in an improved resistance to the deformation. Similarly, when carrot powder was added to the food formula, water absorption increased, leading to a more consistent dough and improved printability (Ahmadzadeh et al. 2024). The valorization of orange peel waste (OPW) in 3D‐printed snacks further illustrates the benefits of incorporating fiber‐rich by‐products to enhance printability. Combining OPW with xanthan gum augmented the ability to bind water and reinforced layer adhesion. Interestingly, the shear‐thinning behavior of such formulations allowed for smooth extrusion, with initial viscosities ranging from 22,500 to 36,900 Pa·s depending on the xanthan gum concentration (ranging from 0.4% to 1.0% w/w) and ensured that the printed structures remained stable during postprocessing (Da Tan et al. 2023; Leo et al. 2022). Additionally, the incorporation of orange by‐products (OBP) into apricot‐based gels increased their elastic modulus (*G*′) to 834–1378 Pa, further stabilizing the printed structures. This improvement enabled a greater structural stability of the printed food structures thereby ensuring the shape to remain stable during postprocesses, such as freeze‐drying (Molina‐Montero et al. 2023). Further evidence of the role of fiber from food by‐product in improving dough printability was given by producing 3D‐printed functional cookies through the addition of grape pomace and broken wheat. Grape pomace, rich in dietary fiber, enhanced the structural integrity of the dough, allowing for better shape retention during and after printing, especially at 6%–8% content, attaining viscosities (at shear rates approximately 1 s^−1^) between 10,000 and 12,000 Pa·s. However, further increase of grape pomace content led to clogging and damaged filaments during material deposition. Notably, the addition of 6% grape pomace combined with broken wheat demonstrated improved extrusion flow and well‐defined cookie shapes, highlighting the synergistic effect of fiber‐rich by‐products in stabilizing the matrix (Jagadiswaran et al. 2021). Sensory evaluation revealed that cookies containing 4% and 6% grape pomace powder were rated acceptable in terms of flavor, whereas those with an 8% inclusion level exhibited pronounced bitterness and an off‐putting color. Consequently, the 6% formulation emerged as the preferred choice for overall sensory quality (Jagadiswaran et al. 2021). Okara with a high‐fiber content up to 44% dry weight (Lee et al. 2021) is another interesting material to be used in 3DFP applications. Particle size significantly influences printability; reducing okara powder particle size below 100 µm prevents nozzle clogging and enhances layer fidelity during extrusion. A reduction in particle size improved the dispersion of okara particles in the mixture, lowering water holding capacity (WHC) and preventing agglomeration, which otherwise would clog the printing nozzle. The WHC of okara decreased from 3.97 g water/g okara for 300–500 µm particles to 2.20 g water/g okara for particles below 100 µm. In addition, the yield stress increased from 42 Pa for particles below 100 µm to 130 Pa for larger particles. Furthermore, the viscosity at rest for the ink with fine okara particles was 2100 Pa·s, which allowed for smooth extrusion and excellent structural retention during printing. The storage modulus (*G′*) for this ink was measured at 4300 Pa, providing the necessary mechanical strength for shape retention after deposition (Lee et al. 2021).

##### 3.3.1.2 Effect of Starch‐Rich FBS Materials

Starch‐rich by‐products are another interesting category of materials with potential in improving the printability of food inks due to their ability to affect rheological properties, promoting better flow behavior during extrusion, and providing structural integrity to the printed materials. For food formulas designed for 3D‐printed noodles, potato peel waste (PPW), with 50.2% starch content, significantly improved the printability of a dough. Fine PPW particles (<0.125 mm) exhibited a high surface area, which allowed for more efficient water absorption, thereby increasing the WHC of the dough. The enhanced WHC was a crucial factor in maintaining moisture throughout the extrusion process, resulting in improved cohesion and stability during printing. In this experiment, the mechanical forces applied during extrusion facilitated the formation of a gel network, which was essential for maintaining the structural integrity of the printed noodles. The developed dough displayed shear‐thinning behavior, with viscosity decreasing from 23,533 to 2.87 Pa·s as the shear rate increased from 0.1 to 100 s^−1^. This rheological behavior enabled the material to flow smoothly through the 0.8‐mm nozzle and extrusion pressure of 0.06 MPa, while it regained viscosity immediately upon deposition, ensuring the noodles retained their shape throughout the printing and postprocessing stages (Muthurajan et al. 2021).

Jackfruit seeds, another starch‐rich by‐product, have shown similar benefits in enhancing the printability of food inks. The high starch content in jackfruit seeds (50%–55%) contributed to the formation of a robust gel matrix during extrusion. Jackfruit seeds were first partially gelatinized followed by grinding to create a paste with appropriate viscosity for the 3DP process. This food‐ink was extruded at 25°C through a 4‐mm nozzle generating a stable network and high‐quality printed structures that retained their shape postextrusion. However, when smaller nozzles (e.g., 1.5 mm) were used, the high viscosity of the jackfruit seed ink led to blockages, highlighting the need for further optimization to improve flowability (Hooi Chuan Wong et al. 2022). Gelatinization and water‐binding capacity of the starch within these by‐products led to improved printability. The starch contributed to the formation of a stable gel network during extrusion, which helped maintain the integrity of the printed structure.

##### 3.3.1.3 Effect of Bioactive‐Rich FBS Materials

The integration of bioactive compounds from food by‐products, such as GSE and GTE, into soy protein isolate (SPI) matrices for 3D‐printed edible packaging have been studied. Protein–polyphenol interactions modified the rheological properties and structural stability of the printed films, offering new avenues for active packaging applications. In this context, SPI served as the primary matrix for fabricating 3D‐printed edible films with polyphenol‐rich GSE and GTE incorporated at concentrations of 1%, 3%, and 5% (w/w, on SPI‐solids basis). The addition of GSE and GTE modified the gelation behavior by affecting protein–protein interactions within the SPI matrix. The proanthocyanidins in GS interfered with the protein network formation, resulting in weaker gels and lower printability. In contrast, GTE showed a more favorable impact on the printability of the SPI matrix. GT‐loaded formulations, particularly those containing 3% GT (SPI–GT3), showed less extent of protein–polyphenol interactions, allowing the gelation process to proceed more effectively. The viscosity of the SPI–GT3 formulation was measured at approximately 2500 Pa·s at low shear rates, which, while lower than the control, was still sufficient to maintain structural integrity during printing. This moderate reduction in viscosity allowed the films to be extruded consistently at a pressure of 0.062 MPa through a 0.25‐mm nozzle, ensuring that the printed films retained their designed shapes with minimal deformation. The better printability of the GT films was attributed to the presence of limited interactions between the polyphenols in GT and the SPI, which did not interfere significantly with gelation (Ahmadzadeh et al. 2023).

##### 3.3.1.4 Effect of Protein‐Rich FBS Materials

The use of PPP, pea protein concentrate (PPC), and SPI in dough formulations highlighted the importance of selecting appropriate protein types and plasticizers for printing optimization. PPP, due to its superior flow characteristics, required lower glycerol content (< 50 wt%) to achieve printable doughs than both PPC and SPI. The best performance was obtained with a formula containing 45% PPP and 55% glycerol with a zero‐shear viscosity of 30 mPa·s, which facilitated smooth extrusion and accurate shape retention. In contrast, both PPC and SPI required higher glycerol levels to achieve comparable flowability. Excessive glycerol led to overly fluid doughs that compromised structural integrity. These findings highlight the delicate balance between protein content and plasticizer levels required to maintain the viscoelasticity needed for successful 3DP (Álvarez‐Castillo et al. 2021). An interesting study utilized SSG to develop 3D‐printed hydrogels for food‐related biomaterials (Carvajal‐Mena et al. 2022). Collagen, the primary component of gelatin, was critical in determining the printability of the hydrogel due to its role in the gelation process. At concentrations of 2% and 5%, the collagen in the gelatin was insufficient to form strong gel networks, resulting in inks with low viscosities (i.e., 200 Pa·s) even at low shear rates. This condition led to an overflow during extrusion and poor shape retention. At higher gelatin concentrations, the collagen interacted and cross‐linked more effectively, significantly improving the material's gelation and structural stability. At 8% gelatin, the viscosity increased to 1200 Pa·s, allowing the ink to be extruded smoothly while maintaining its shape after printing; the stronger gel networks also provided an optimal balance between flowability and structural integrity. Further increase in gelatin concentration (e.g., 14%) resulted in a viscosity of 3500 Pa·s, which required higher extrusion pressures, from 0.02 to 0.05 MPa, to maintain consistent flow. However, the ability of collagen to form strong, temperature‐dependent gel networks was essential to maintain the printing fidelity of the hydrogels, making SSG a highly effective material for 3DFP applications.

The development of 3D‐printed surimi was realized by leveraging the high protein content of cod by‐products. The conventional washing method yielded surimi with better printability compared to the pH‐shift processing method. In the conventional washing method, the addition of salt at 1.5% or 3% facilitated myofibrillar protein swelling and WHC (up to 4.12 g of water per gram of surimi), which improved flow behavior, gel strength, and cohesiveness during printing. This increase in WHC was crucial for maintaining the viscosity and gelation of the surimi during printing, ensuring the material retained its shape postextrusion (Gudjónsdóttir et al. 2019). In addition, salt induced better gel formation, stabilizing the structure of the surimi and allowing for smoother flow through the nozzle. Low‐field nuclear magnetic resonance (LF‐NMR) was used to analyze water distribution and protein–water interactions during processing and revealed that 3% salt significantly reduced the most restricted water population (T21 of 59.4 ms, A_21_ = 89.5%), while the intermediate population (T22 of 97.7 ms, A_22_ = 6.8%) increased, indicating better water retention and gel network stability in the conventionally washed surimi. In contrast, the pH‐shift process which consists of alkaline protein solubilization (pH 11) followed by acid precipitation (pH 5.5), often results in the aggregation of proteins, particularly myofibrillar proteins, which interferes with smooth extrusion. These aggregated proteins led to a stickier surimi paste and a more gel‐like texture, which resulted in gaps between deposited layers during printing (Gudjónsdóttir et al. 2019).

#### 3.3.2 The Use of FBS Materials to Enhance the Nutritional Content of Food Inks

##### 3.3.2.1 Through Dietary Fiber Fortification

**FIGURE 6:**
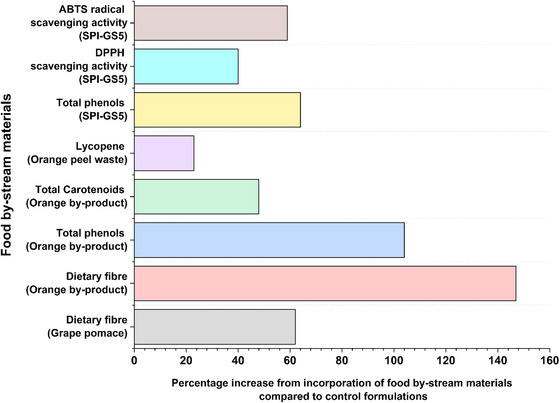
Illustration of the percentage increase in key nutrients achieved by incorporating various food by‐stream materials into 3D‐printed foods.

The incorporation of fiber‐rich food by‐products such as grape pomace and OBP into 3D‐printed food formulations significantly enhances dietary fiber content, potentially offering a personalized approach to addressing fiber deficiencies in modern diets (Figure 6). Dietary fiber, particularly insoluble fiber, plays a vital role in promoting gut health and reducing the risk of gastrointestinal disorders such as diverticulosis and colorectal cancer (Jagadiswaran et al. 2021; Muthurajan et al. 2021). In one study, the incorporation of grape pomace, a by‐stream material rich in dietary fiber, into cookie formulations significantly enhanced their nutritional profile. Specifically, the addition of 15% grape pomace increased the dietary fiber content by 62%, from 3.76% in the control cookies to 6.09% in the fortified cookies. This fortification not only contributed to higher fiber intake but also improved the functional properties of the dough, including its water absorption capacity and structural stability during baking (Jagadiswaran et al. 2021). Similarly, the incorporation of OBP into 3D‐printed apricot gel snacks led to a substantial improvement in dietary fiber content. The fiber content increased from 1.31 g/100 g in the control formulation to 3.24 g/100 g in the OBP‐fortified product, representing a 147.3% increase. This enhancement aligns with the nutritional claims requirements of the European Union, significantly boosting the health benefits of the printed snacks (Molina‐Montero et al. 2023).

##### 3.3.2.2 Bioactive Ingredients Delivery via Food By‐Product Integration

Adding bioactive compounds from food by‐products, such as OBP, GSE, and GTE, provides personalized health benefits through the targeted enhancement of antioxidant capacity (AC) and the reduction of oxidative stress. For example, incorporating OBP into 3D‐printed apricot pulp snacks significantly enhanced their antioxidant profile. The total phenol (TP) content, a key indicator of antioxidant activity, increased from 168 mgGA/100 g in the control formulation (FD70, freeze‐dried sample with 70% apricot pulp) to 344 mgGA/100 g in the OBP‐enriched formulation (FDBP70, freeze‐dried 70% apricot pulp with 3.56% OBP), representing a 104.8% increase. Similarly, the total carotenoid (TC) content, another vital antioxidant component, increased by 47.9%, from 4.8 mgβ‐carotene/100 g (FD70) to 7.10 mgβ‐carotene/100 g (FDBP70). The lycopene (LP) content also rose by 22.9%, while the AC, as measured by Trolox equivalents, showed a marginal improvement from 99 mgTrolox/100 g (FD70) to 101 mgTrolox/100 g (FDBP70). These enhancements underscore the potential of OBP to boost the nutritional and functional properties of 3D‐printed apricot pulp snacks, providing a richer source of antioxidants that help combat oxidative stress and support overall health (Molina‐Montero et al. 2023). The OPW‐based 3D‐printed snacks demonstrated significantly enhanced antioxidant profile. OPW is rich in bioactive flavonoids such as hesperidin (476.0 ± 8.6 mg/100 g) and narirutin (241.3 ± 14.4 mg/100 g), both of which contribute to potent antioxidant activities. These compounds remained stable throughout the stages of food printing, from powder to ink to the final printed product. The AC, assessed through DPPH and ABTS assays, showed that the antioxidant properties of OPW were unaffected by the 3DP process (Da Tan et al. 2023). A similar study demonstrated the significant antioxidant potential of GSE and GTE. GS exhibited superior antioxidant properties, with a total phenolic content (TPC) of 1800 µg GAE/g extract, which was 80% higher than GT's 1000 µg GAE/g extract. GSE achieved complete DPPH radical inhibition (≈ 100%), about 10% higher than that of the GTE, confirming the superior antioxidant potency of GS. Similarly, GS achieved a 25% higher ABTS radical scavenging activity, at 7500 µg GAE/g extract, compared to GT's 6000 µg GAE/g extract. For ferric reducing antioxidant power (FRAP), GS recorded 550 µg GAE/g extract, representing an 83.3% increase over GT's 300 µg GAE/g extract. When incorporated into SPI films, the antioxidant benefits of GS and GT were further reflected. Films containing 5% (SPI‐GS5) exhibited a TPC of 10 µg GAE/g film, which was 64% higher than no extract (SPI‐C) films (6.1 µg GAE/g film). Films containing 5% (SPI‐GT5) exhibited a TPC of 11 µg GAE/g film, which was 80.3% higher than SPI‐C films (Figure 6). Similarly, DPPH scavenging activity in SPI‐GS5 films was 57%, a ∼2.4‐fold (≈ 137%) enhancement over the SPI‐C control (27%) and in SPI‐GT5 films was 48%, showing a 77.78% improvement over SPI‐C films. The ABTS radical scavenging activity was also elevated by 100%, with 20 µg GAE/g film for SPI‐GS5 compared to 10 µg GAE/g film for SPI‐C and a 50% increase with 15 µg GAE/g film for SPI‐GT5 when compared with SPI‐C films. Lastly, FRAP values increased by 600%, with SPI‐GS5 films achieving 7 µg GAE/g film versus 1 µg GAE/g film for SPI‐C and an increase of 400%, with 5 µg GAE/g film for SPI‐GT5 versus SPI‐C film. These results underscore GS's superior capacity to enhance the antioxidant properties of both extracts and their respective films, making them ideal for functional food applications (Ahmadzadeh et al. 2023).

##### 3.3.2.3 Protein Enrichment for Specific Dietary Needs

Certain populations—such as older adults, athletes, and individuals recovering from illness—have protein requirements that exceed the 0.83 g/kg body weight/day reference level for healthy adults; for example, older adults often benefit from intakes of 1.0–1.2 g/kg body weight/day to preserve muscle mass, and clinical patients may require up to 1.5 g/kg body weight/day (Deutz et al. 2014; EFSA 2012). 3DFP enables targeted fortification by precisely incorporating high‐quality proteins into customized matrices, thereby meeting these specific requirements without compromising texture or palatability. Protein fortification through the use of food by‐products such as SGG, and PPP allows for the creation of personalized high‐protein food products such as functional hydrogels. SGG, derived from fish processing waste, was shown to enhance the protein content of 3D‐printed gelatin hydrogels, which were developed for use as biomaterials in food‐related applications. SGG formulations containing 8% gelatin exhibited a 98.14 g/100 g of SGG protein content, making them suitable for individuals requiring higher protein intake, such as athletes or those recovering from illness. The gelatin also contributed to the formation of a strong gel network, which improved the texture and mouthfeel of the final product while delivering essential proteins (Carvajal‐Mena et al. 2022). PPP, when partially replaced by PPC, enhanced the protein profile of 3D‐printed snacks. The biopolymer formulation, containing up to 10% PPC within a 45% biopolymer fraction, exhibited protein contents of 74.0% for PPP and 88.0% for PPC, significantly enriching the overall protein content. This combination provided a balanced source of essential amino acids, making it ideal for individuals with increased protein requirements (Álvarez‐Castillo et al. 2021).

##### 3.3.2.4 3D‐Printed Soft Foods for Dysphagia Management

The valorization of spinach stems and kale stalks was attained by developing 3D‐printed soft foods designed to meet the International Dysphagia Diet Standardization Initiative (IDDSI) standards (Pant et al. 2023). Specifically, the printed foods adhered to the IDDSI Level 4 standards, classifying them as “pureed” foods appropriate for patients with dysphagia. However, the low initial viscosity of the spinach and kale purees compromised the structural integrity of the printed shapes. To address this, binding agents, such as potato starch, sweet potato puree, and xanthan gum, were introduced. These components improved printability by enhancing textural firmness while maintaining the necessary softness for easy swallowing. The optimal formulation, consisting of 30% spinach stem puree, 20% kale stalk puree, 35% sweet potato puree, and 15% potato starch, provided a balance between flowability during extrusion and postprinting stability. In overall, the inclusion of these ingredients ensured sensory acceptability by dysphagia patients. Consistent flow and successful creation of the IDDSI‐compliant food shapes were achieved using an extrusion pressure of 0.04–0.06 MPa and a 1.2‐mm nozzle size (Pant et al. 2023).

The enhancement of probiotic viability in soft, dysphagia‐friendly foods was achieved by creating 3D‐printed formulations enriched with *Lactiplantibacillus plantarum* and stabilized by WPI nanofibrils through biointerfacial supramolecular self‐assembly (Zhang et al. 2024). Specifically, the WPNFs coating provided stability against environmental stresses encountered in 3DP, aligning with the IDDSI Level 5 standards (“minced and moist”) suitable for dysphagia patients. Initial trials highlighted that, without structural support, the probiotics’ viability and shape retention postprinting were compromised due to mechanical shear and extrusion conditions. However, incorporating high acyl gellan gum (HG), fructooligosaccharides (FOS), and WPI enabled optimized extrusion properties while maintaining adequate softness. An ideal composition of 0.25 g HG, 1.25 g WPI, 0.75 g FOS, and 3.75 mL Lp‐WPNFs–GTB emulsion provided balance between mechanical strength for shape retention and softness required for safe swallowing. The extrusion‐based 3DP was conducted at 45°C using a nozzle diameter of 0.84 mm and a print speed of 20 mm/s, producing stable probiotic‐rich structures with a viable cell count exceeding 8.0 log CFU/g assembly (Zhang et al. 2024). Sensory and IDDSI testing confirmed the acceptability of the printed shapes, ensuring their suitability as safe, nutrient‐enriched foods for dysphagia management.

#### 3.3.3 The Use of FBS Materials to Maximize the Sensory Properties of 3DP Foods

##### 3.3.3.1 Color Enhancing Compounds

**FIGURE 7:**
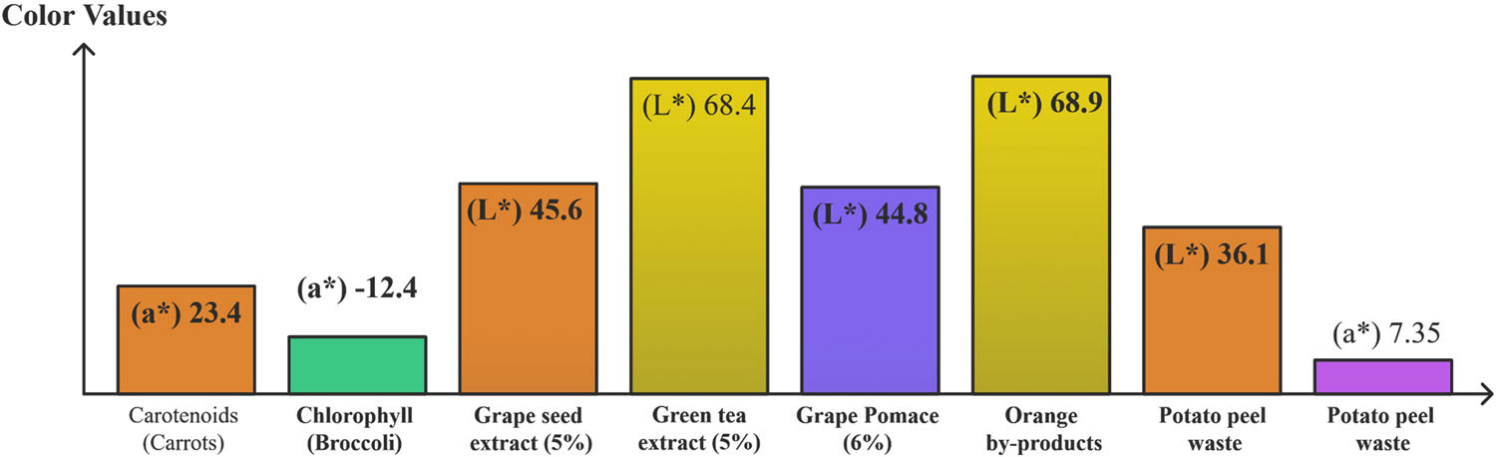
The color properties (L*, a*, b* values) of 3D‐printed foods enhanced with natural pigments from food by‐stream materials. GSE, grape seed extract; GTE, green tea extract; OBP, orange by‐products; PPW, potato peel waste.

Color determines the sensory appeal of a food, and it is primarily conferred by the natural pigments present in the ingredients. In 3D‐printed snacks, utilizing vegetable by‐products poses an additional advantage; pigments such as carotenoids and chlorophyll in the ingredients contribute significantly to their color profile (Figure 7). For example, adding carrots, rich in carotenoids, resulted in a distinct reddish‐orange hue in uncooked wheat flour‐based snacks, with a redness (a*) value of 23.4. Similarly, the presence of chlorophyll in broccoli produced a green coloration in the uncooked snacks, with a negative a* value of −12.4. However, after baking at 177°C for approximately 8 min, a regime chosen to set structure without over‐drying, these pigments underwent degradation, leading to a reduction in color intensity, demonstrating the inherent limitations of maintaining vibrant hues during thermal processing (Ahmadzadeh et al. 2024). Incorporating polyphenol‐rich by‐products such as GSE and GTE significantly modified the color of 3D‐printed edible films too. The polyphenols in these extracts reduced the lightness (L* values) of the films. For instance, SPI films containing 5% GSE had L* values about 45.6, while films with 5% GTE exhibited lighter hues (L* = 68.4). These polyphenols, known for their light‐scattering properties, effectively reduced the brightness of the films, making them suitable for packaging applications for light‐sensitive products (Ahmadzadeh et al. 2023). Grape pomace, another polyphenol‐rich by‐product, played a similar role in the creation of 3D‐printed cookies. Enzymatic browning caused by polyphenol oxidase in grape pomace led to a darker color, with cookies containing 6% grape pomace showing a lower L* value than their respective control (without pomace) (44.8 vs. 52.6). Browning was further enhanced by Maillard reactions and caramelization during baking, contributing to a rich, brown hue that enhanced the visual appeal of the cookies (Jagadiswaran et al. 2021).

OPW addition also improved the color of 3D‐printed snacks. The high concentration of bioflavonoids, particularly hesperidin and narirutin, in OPW contributed to a darker color in the final product. Hesperidin was found at levels of 476.0 ± 8.6 mg/100 g, and narirutin at 241.3 ± 14.4 mg/100 g in the dried OPW, providing the printed snacks with a distinct yellow‐orange hue. The bioflavonoids were effectively retained throughout the 3DP process, emphasizing their stability and contributing to the visual characteristics of the product (Leo et al. 2022). Similarly, in another study, revalorizing OPW through DIW technology further showcased the role of bioflavonoids in enhancing the appearance of printed snacks. The yellow‐orange hue observed in the final product was largely due to the presence of hesperidin and narirutin, which contributed to the color and were also preserved during printing. These compounds remained stable, with hesperidin measured at 476.0 ± 8.6 mg/100 g and narirutin at 241.3 ± 14.4 mg/100 g, ensuring a consistent appearance in the printed snacks (Da Tan et al. 2023). Additionally, the inclusion of OBP in the formulation of apricot gel snacks significantly enhanced the brightness and color saturation of the printed gels. Samples containing OBP exhibited increased brightness, as evidenced by their higher L* values than those of the control, 68.9 vs. 55.5. OBP also heightened the yellowish hue (b* values) while reducing the reddish hue (a* values), resulting in a more visually appealing product (Molina‐Montero et al. 2023). Lastly, the incorporation of PPW into 3D‐printed noodles provided a distinctive brown color due to the high polyphenol content in the peel. Enzymatic browning during processing reduced the lightness (L*) of the noodles from 66.7 (commercial samples) to 36.1. Simultaneously, the polyphenols contributed to a higher redness (a* = 7.35), further distinguishing the appearance of the noodles from traditional counterparts (Muthurajan et al. 2021).

#### 3.3.4 Texture Enhancement Using Printing Process Parameters and FBS Materials

**FIGURE 8:**
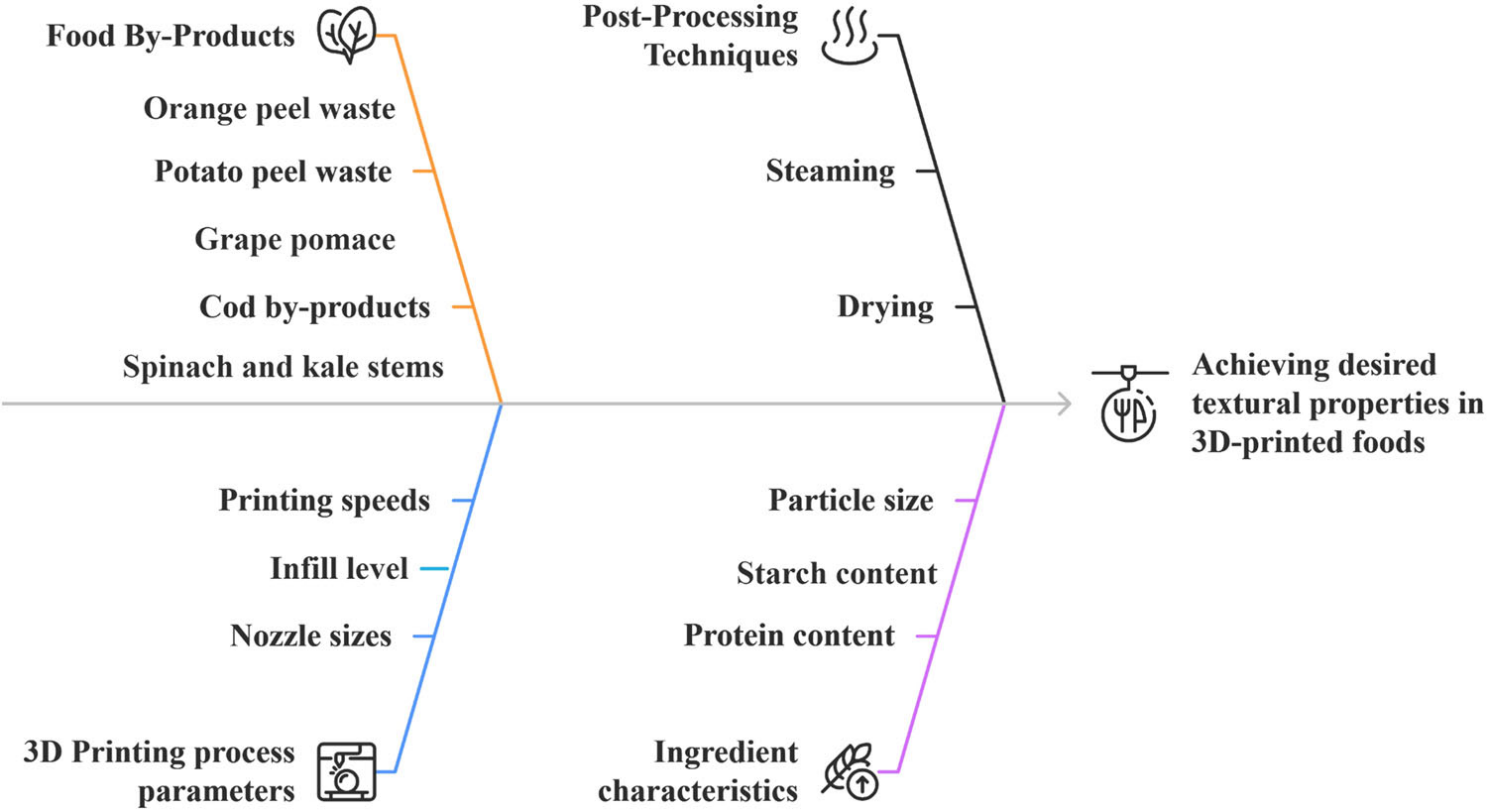
The contributions of food by‐products and food waste, 3D printing process parameters, postprocessing techniques, and ingredient characteristics to achieving desired textures.

Textural properties of 3D‐printed foods are often enhanced through the integration of food by‐products; however, this outcome depends on the precise selection of printing process parameters. By‐products like vegetable powders, fish proteins, starches, and fiber‐rich materials contribute to structural and functional properties that can be optimized through process controls such as extrusion pressure, nozzle size, and postprocessing (Figure 8). A significant impact on texture was observed when PPW was used in noodle production. Larger nozzle sizes facilitated the creation of thicker layers, contributing to firmer noodle structures. Three nozzle diameters, namely 0.50, 0.82, and 1.28 mm, were screened; the largest (1.28‐mm nozzle, chosen to attain the ≈ 1.7 mm commercial strand thickness) produced thicker layers and, after steaming + drying, the highest noodle firmness, whereas the 0.50–0.82‐mm nozzles yielded thinner, less rigid strands. Additionally, slower printing speeds improved layer adhesion, reducing surface stickiness. Postprocessing, including steaming and drying, often reduces moisture content, making the noodles firmer and more stable. This showcases how PPW and precise parameter control can synergistically enhance the textural properties of 3D‐printed noodles (Muthurajan et al. 2021). Cod by‐products valorization through surimi ink production provides another example of tailoring process parameters for texture enhancement. When using the conventional washing method combined with salt concentrations of up to 3%, the printed surimi displayed increased gel strength, firmness, and cohesiveness. Salt‐induced swelling of myofibrillar proteins increased water retention, which was crucial for maintaining texture during printing and postprocessing. Salt level selection and washing methods played a critical role in achieving desirable texture outcomes in 3D‐printed surimi (Gudjónsdóttir et al. 2019). In okara‐based 3DP, texture was modulated by adjusting okara particle size and infill levels. Fine particle sizes led to improved smoothness and better extrusion consistency, resulting in a more cohesive and firmer final product. Higher infill levels provided additional structural integrity and hardness, indicating that process parameters like particle size reduction and infill can be optimized to create a range of textural profiles (Lee et al. 2021). The use of grape pomace and OPW further illustrates the role of food by‐products in texture enhancement. Grape pomace reduced the hardness of printed cookies, creating a softer yet stable texture, while OPW improved the firmness of printed snacks by stabilizing the ink formulation and promoting consistent extrusion. The postprinting drying of OPW‐based snacks solidified their texture, demonstrating the importance of combining food by‐products with controlled postprocessing techniques to achieve desired textural qualities. Spinach stems and kale stalks, combined with starch‐rich ingredients like potatoes, were utilized to produce soft foods tailored for dysphagic patients. By adjusting the ratio of vegetable and starch components, firmness and cohesiveness of the printed foods were controlled so that they were soft enough for consumption while maintaining structural integrity (Pant et al. 2023). Rheological adjustments, by using hydrocolloids like gelatin, further enhanced the ability to achieve specific textural properties required for medical food applications. In surimi production, the pH‐shift method introduced additional complexities in texture control. While the method recovered proteins effectively, the aggregation of the myofibrillar proteins led to a stickier paste with lower cohesiveness compared to the conventional method. The modulation of pH, combined with optimized salting, allowed partial mitigation of these issues, improving the firmness and printability of surimi. Furthermore, the incorporation of protein‐rich by‐products, such as SSG, into food inks showcased how collagen content contributed to texture enhancement (Carvajal‐Mena et al. 2022). The capacity of collagen to form gel networks at higher concentrations significantly improved mechanical stability and firmness of printed structures, particularly at concentrations above 8% gelatin. The resulting textural improvements allowed proper modulation of extrusion pressure and temperature, which were critical in maintaining shape retention and firmness after printing. Across these studies, the effective utilization of food by‐products for texture enhancement hinges on a combination of ingredient characteristics and process parameters. Whether leveraging fiber‐rich materials, adjusting protein content, or optimizing particle size and extrusion techniques, the controlled use of food by‐products allows for the fine‐tuning of textures in 3D‐printed foods. These processes not only enhance sensory characteristics but also contribute to the sustainability of food production by incorporating otherwise discarded materials into value‐added products.

### 3.4 Role of 3DP FBS Materials in Achieving CE Objectives and Global Sustainability Agendas

**FIGURE 9:**
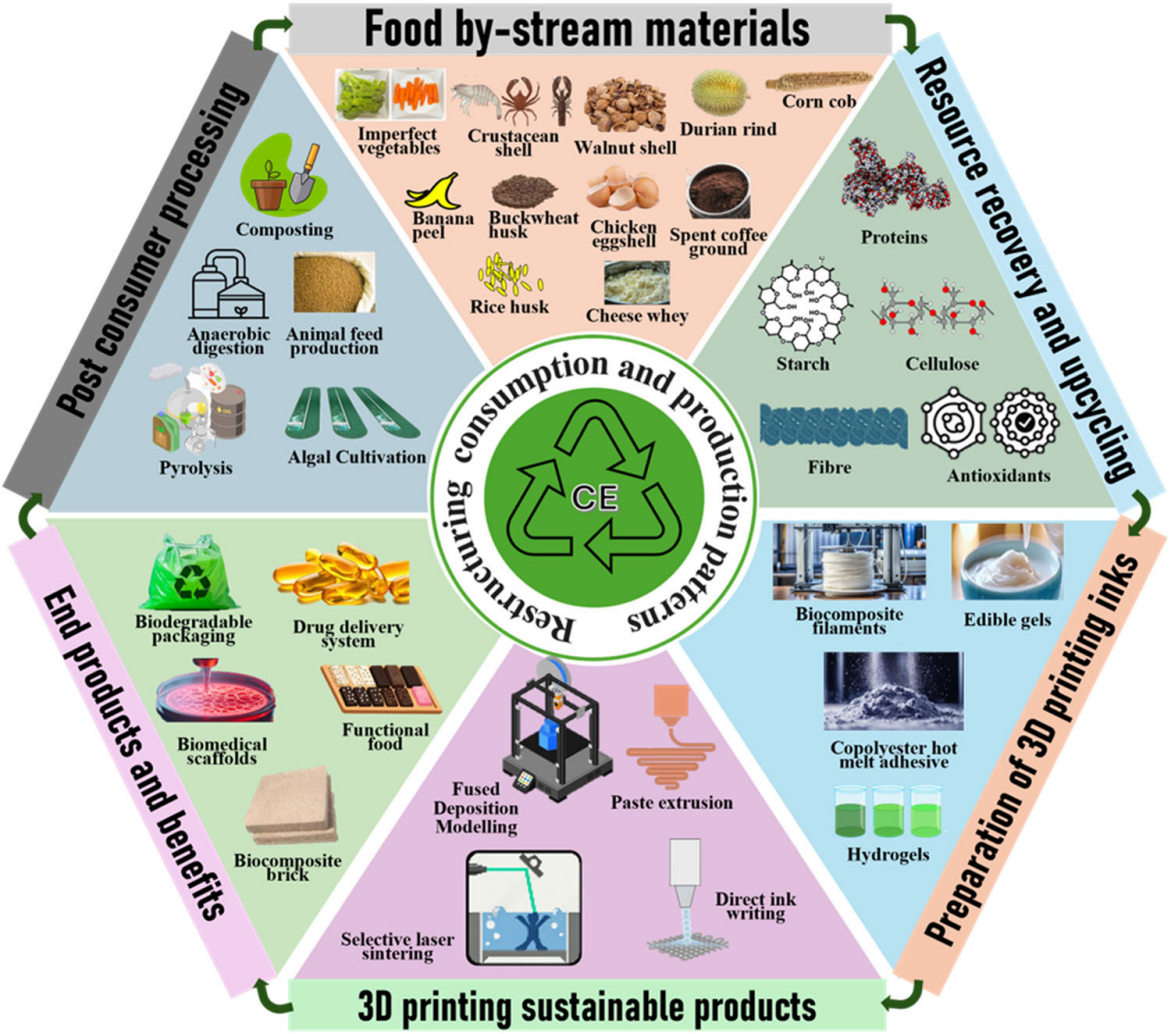
Circular economy framework for 3D printing with food by‐products, illustrating six interconnected stages. The process starts with the collection of diverse food by‐products, followed by resource recovery to extract valuable components. These are used to prepare 3D printing inks, enabling the fabrication of sustainable products through various 3D printing techniques. The end products include biodegradable packaging and functional foods. Postconsumer processing methods like composting and pyrolysis ensure material regeneration, closing the loop by feeding back into the collection stage.

3DP of food by‐products is pivotal in achieving CE objectives by establishing a closed‐loop system across six interconnected stages (Figure 9). The cycle starts with the collection of food by‐products, such as banana peels, walnut shells, and rice husks, which are otherwise considered waste and discarded or significantly underutilized. This otherwise waste and its components undergo recovery and upcycling, where valuable nutrients and materials like proteins, cellulose, and antioxidants are extracted for further utilization. The extracted components are then used in 3DP inks, enabling the development of specialized materials, such as biocomposite filaments and edible gels. Consequently, 3DP (FDM, paste extrusion, and SLS) incorporating these upcycled components transforms these inks into a wide range of sustainable products, such as biodegradable packaging, functional foods, biomedical scaffolds, and biocomposite bricks. The end products and benefits demonstrate the diverse applications that contribute to reducing the consumption of virgin resources. To complete the circular flow, postconsumer processing methods, including composting, anaerobic digestion, and pyrolysis, facilitate the recycling or repurposing of end‐of‐life products. This phase allows for nutrient recovery and material regeneration, feeding back into the initial stage of by‐product collection. By integrating these stages, 3DP of food by‐products aligns with CE principles, restructuring consumption and production patterns to achieve sustainable material reuse, minimizing waste, and closing the loop in resource utilization. The integration of food by‐products into 3DP also offers significant potential for advancing CE objectives, described as following.

#### 3.4.1 Lower Emissions in Postconsumer Processing

One of the benefits of using food by‐products in 3DP is their potential for energy recovery with lower carbon emissions. The carbon‐neutral aspect of food by‐product recovery lies in the fact that the carbon emitted during decomposition or energy recovery was originally absorbed by plants during growth. This contrasts sharply with synthetic materials like plastics, which release significant amounts of CO_2_ and other pollutants when incinerated. This review emphasizes the wide range of food by‐products that can be repurposed not only into functional food products. SCG/PLA 3D‐printed composites showed higher thermal stability (by 10°C–15°C) than pure PLA, making it suitable for energy recovery applications after its product lifecycle (Romani et al. 2024). Thermogravimetric analysis confirmed that incorporating 10 wt% SCGs delays the 5% weight‐loss onset temperature (*T*_5_%) by ≈ 10°C–15°C—for example, from 407°C to 417°C for rLDPE and from 410°C to 422°C for HDPE—indicating a tangible improvement in thermal stability. Similarly, rice husk/PLA composites can withstand temperatures up to 300°C, making them ideal for high‐heat applications such as fire‐resistant building components (Morales et al. 2021a, 2021b). These materials can then be processed into bioenergy without contributing to harmful emissions.

#### 3.4.2 Localized Upcycling of Perishable Food Waste for On‐Demand Manufacturing

Perishable food waste presents a unique challenge due to its quick decomposition and methane emissions when left untreated. By incorporating food by‐products into local 3DP, industries can mitigate these emissions by reducing the need for transportation and centralized waste processing. A significant step forward in this area is the use of perishable waste such as vegetable stalks, fruit peels, and fish bones at their generation site to produce 3D‐printed products locally. Incorporation of OPW to create biodegradable packaging can reduce environmental impact of both food waste and plastic packaging as demonstrated in literature (Leo et al. 2022; Molina‐Montero et al. 2023). Fish bone waste could be transformed into biocomposites for biomedical applications, leveraging the calcium carbonate content to produce high‐strength, biodegradable materials (Scaffaro et al. 2022). These examples showcase how locally used 3DP can upcycle food waste into functional products, reducing the overall carbon footprint by eliminating the need for long‐distance transportation and preventing methane emissions from decomposing waste.

#### 3.4.3 Customizable Nutrient‐Dense Foods for Sustainable Food Production

3DP offers unique opportunities to create customized, nutrient‐dense food products from food by‐products, contributing to a more sustainable food production system. By upcycling waste materials like okara or grape pomace into 3D‐printed snacks, the food industry can reduce food waste while producing nutritionally enhanced foods. Okara 3D‐printed snacks could carry 33% of okara and endow these products with protein content of 28%, significantly enhancing their nutritional value (Lee et al. 2021). Similarly, grape pomace enriched 3D‐printed cookies, demonstrated boosted antioxidant content and fiber by up to 8% (Jagadiswaran et al. 2021). Incorporating food‐industry by‐products into 3D‐printed foods has been shown to divert large fractions of material from waste streams. A techno‐economic assessment found that using waste‐derived inks can reduce overall food‐waste disposal by 20%–30% compared to conventional processing techniques (Yu and Wong 2023). Food waste conversion into printable materials can achieve reductions in waste volumes of 40%–60% (Pant et al. 2023; Yoha and Moses 2023). These studies highlight the potential for food waste to be repurposed into nutritionally dense, customizable food products, providing an added layer of sustainability.

#### 3.4.4 Reduction of Agricultural Input for Secondary Products

One of the significant advantages of 3DP with food by‐products is the reduction in the need for additional agricultural inputs to produce secondary products like packaging, bioplastics, or construction materials. By repurposing food waste into these products, the food and related industries can minimize carbon emissions and resource use associated with growing new crops specifically for these purposes. For example, combining wheat gluten and keratin from animal by‐products to produce bioplastics with tensile strengths of 13.5 MPa, suitable for biodegradable packaging applications, allow reducing the reliance on virgin materials (Alshehhi et al. 2024).

#### 3.4.5 Contribution of 3DP FBS Materials to Global Sustainability Goals

The integration of FBS materials into 3DFP offers a novel approach to advancing several global sustainability objectives, particularly those outlined in the United Nations SDGs. By transforming nutrient‐rich residues such as fruit pomace, vegetable peels, or cereal bran into printable food matrices, this strategy directly supports SDG 12 (Responsible Consumption and Production) by promoting waste valorization and minimizing resource loss in the agri‐food chain. It also contributes to SDG 2 (Zero Hunger) through the creation of customizable, nutrient‐enhanced foods suitable for malnourished or vulnerable populations, especially when paired with personalized nutrition strategies. Moreover, decentralized production using 3DFP reduces food miles and refrigeration requirements, aligning with SDG 13 (Climate Action) by lowering emissions associated with conventional food processing and transport. From a policy standpoint, the European Green Deal and the FAO's food loss prevention framework both emphasize circular resource use and technological innovation as levers for food system sustainability. In this context, 3DFP using FBS complements policy tools by creating high‐value applications for materials that would otherwise contribute to landfill or energy‐intensive disposal. Although still in its early stages, this application of 3DP exemplifies a transition toward circular, resilient food systems that couple environmental stewardship with nutritional innovation. Further alignment with regulatory frameworks and life‐cycle metrics will be critical to scale its impact within broader sustainability agendas.

## 4 Future Scope and Perspectives

### 4.1 Improvements in 3DFP Incorporating FBS Materials

Coaxial extrusion is an innovative technique in 3DP that allows for the simultaneous deposition of two distinct materials via a specialized nozzle. This technique is particularly valuable for creating multilayered or encapsulated structures, often used to protect bioactive compounds during the printing process, ensuring that sensitive ingredients retain their functionality. However, to this date, coaxial extrusion has not been applied to food by‐products (Cotabarren et al. 2023; Srivastava et al. 2023). Similarly, 4D food printing introduces a dynamic aspect to printed food structures, enabling them to alter shape, texture, or flavor over time when exposed to stimuli such as temperature, moisture, or pH changes. This approach could be particularly useful for developing foods that transform during storage or consumption, such as foods that gradually release flavors or nutrients as they are chewed or heated. Although this application offers exciting prospects such as noodles that curl when boiled, chocolates that change color and then release a second flavor note, or snacks that soften and release nutrients in the mouth for novel consumer experiences and functional foods, no studies covering food by‐products have employed 4D printing to this date. This highlights another significant area for future exploration, where 4D printing could be utilized to further enhance the adaptability and functionality of food by‐products in 3D‐printed foods (Sundarsingh et al. 2023).

A deeper investigation into the potential of functional geometries to influence the sensory and nutritional experience of food is another area of potential of using food by‐products in 3DFP. Advances in computational design tools allow for the creation of intricate internal structures, such as porous matrices or lattices, that can control the release of nutrients, flavors, or even textures during consumption. For example, certain geometries could be designed to break down at different rates in the mouth, enhancing the sensory experience by delivering bursts of flavor or nutrients at specific moments (Cotabarren et al. 2023; Sundarsingh et al. 2023) This digital design approach could be further integrated with food by‐products, optimizing both the consumption experience and the nutritional content of 3D‐printed foods.

### 4.2 Dairy Waste Valorization

The use of dairy by‐products for nonfood 3DP may raise ethical concerns and suggests the need to develop new strategies to ensure more sustainable practices. In this context and considering the annual global waste of dairy products accounting for over 220 million tons (Cecilia et al. 2019), to recover proteins from expired milk may represent a promising strategy for future research toward the development of sustainable nonfood 3DP applications. Fluid milk is estimated to account for approximately 67% of total dairy food loss by weight in the United States, with pasteurized milk being the largest contributor among drinking milk types due its perishability. During its shelf life, lasting 7–14 days in the refrigerator, pasteurized milk undergoes chemical, physicochemical, and microbiological changes, with the most common issue being the development of off‐flavors induced by enzymes and light exposure (D'Incecco et al. 2024). After reaching its expiration date, pasteurized milk is classified as “special category 3 waste,” making it no longer suitable for human consumption. However, expired milk still contains valuable proteins, that is, 27 g/L of casein and 6 g/L of whey proteins, as well as 36 g/L of fat that could be upcycled for the production of biodegradable and functional biomaterials or used as ink for nonfood 3DP applications (Gerna et al. 2023). Given their significant functional properties, milk proteins are by far the most valuable component to recover from expired milk. However, milk fat, organized in the form of triglyceride globules surrounded by a membrane, may also offer benefits, particularly as a lubricant during the 3DP process. Casein can be recovered through enzymatic or acid coagulation, while whey proteins can be obtained via acid‐thermal coagulation. Both protein types can also be recovered using dedicated flow‐filtration processes. While recovering proteins, different amounts of fat could be retained, depending on the adopted conditions. After recovery, the dehydration process concentrates the components into a powder. Once rehydrated, the viscosity of the resuspended powder can be adjusted by controlling the water‐to‐dry matter ratio, resulting in a stable, extrudable ink that can also be combined with other ingredients to create a composite particularly suitable for extrusion‐based 3DP. This approach offers the additional advantage of precise control over the texture and structure of the printed objects, which is essential for developing personalized solutions. These protein‐based composites can be further explored for their potential in creating biodegradable and functional materials, such as biofilms and hydrogels, which hold promise for applications in tissue engineering, wound healing as scaffolds for cell proliferation, thermoplastics, and packaging materials.

### 4.3 Safety Risk and Mitigation Strategies

The scientific literature on the use of FBS materials in 3DFP has so far concentrated on improving printability, postprinting structure stability and nutrition. Thus, the safety aspects have attracted far less examination. FBS materials are often sourced from heterogeneous waste streams that can carry sizable loads of several pathogens such as *Escherichia coli*, *Staphylococcus aureus*, *Salmonella enterica*, *Listeria monocytogenes*, or human‑norovirus. Limited studies have reported on survival of pathogens or their surrogates during and after 3DP. A dough containing 20% dried apple pomace was inoculated with ≈ 4 log PFU/g Tulane virus, extruded at 25°C through a 1.5‐mm nozzle and stored either at 4°C or 20°C. Viral counts declined <0.2 log at refrigeration temperatures but fell by three log cycles after 96 h at 20°C, indicating that extrusion alone is nonlethal and that ambient storage can contribute to viral inactivation but only over multiple days (Hamilton and Gibson 2024). Studies focused on processing confirmed that extrusion alone rarely exceeds 60°C, thus, 3DP will rarely result in significant microbial load reductions. Cross‑contamination can compound the problem. Pathogen‑transfer experiments involving *S. aureus* and *E. coli* further showed that low barrel temperatures and high travel speeds increase carry‑over from contaminated stainless‑steel capsules to successive batches, demonstrating that operational parameters influence cross‑contamination as much as ingredient quality (Ekonomou et al. 2024). Printer hygiene is an additional weak point; a recent evaluation of manufacturer cleaning protocols for reusable food‑ink capsules found that recommended rinsing failed to eliminate either aerobic plate counts or *S. enterica* when biofilms had formed, indicating the need for validated sanitation cycles or single‑use liners (Hamilton and Gibson 2023). One promising counter‑measure is the application of atmospheric‑pressure cold‑plasma coatings: PLA coupons plasma‐treated for 30 s showed a 3.4 log CFU/cm^2^ decline in *Listeria innocua* biofilm density after 24 h at 25°C, with no observable loss of dimensional accuracy in printed cereal‑bar matrices (Muro‐Fraguas et al. 2020). Importantly, no published investigation has yet examined microbial fate in inks formulated with FBS materials, leaving the safety of these promising substrates unverified. Future research should (1) map contamination routes in FBS material based 3DFP using HACCP methodology, (2) establish and validate lethal time–temperature combinations for the most heat‑resistant pathogens likely to be present, (3) integrate complementary nonthermal hurdles such as cold plasma or UV‑LED that preserve heat‑labile bioactives, (4) redesign consumer‑scale printers with easily sanitized or disposable food‑contact modules, and (5) validate cleaning‑in‑place or single‑use cartridge designs and protocols. Addressing these gaps will ensure that the circular‑economy benefits documented throughout this review translate into safe, market‑ready foods. Beyond microbiological hazards, FBS materials in 3DFP inks may introduce chemical contaminants that require formal risk assessment. Many agricultural by‐products accumulate mycotoxins, particularly aflatoxins and fumonisins, at concentrations capable of exceeding regulatory limits (WHO 2023; Alshannaq and Yu 2017). Heavy‑metal carry‑over is another concern: dried coffee grounds, rice husk ash, and crustacean shells have been shown to contain Pb, Cd, or As at levels that would necessitate exposure assessments if incorporated at ≥ 10% w/w in edible inks (Hassan et al. 2021). Even where the residue itself is compliant, printer hardware can become a secondary source of exposure; brass or stainless‑steel nozzles release Ni, Cr, and Cu when operated under acidic‑ink conditions, and dermal or oral exposure models predict intakes that approach tolerable daily limits for Ni after prolonged use (Wade et al. 2023). Thermal postprocessing can provide a further layer of complexity and concern; it was estimated that shape‑optimized biscuits printed from wheat‑bran dough could exhibit up to 75% higher acrylamide content when baked at 190°C than their conventionally molded counterparts, underscoring the need to control internal geometry, water activity and time–temperature profiles (Abedini et al. 2024; Derossi et al. 2024). These observations signal the need for comprehensive residue screening (mycotoxins, pesticides, heavy metals), migration studies that link printer‑material chemistry to ink pH and fat content, and modeling of process contaminants (acrylamide, HMF, furans) under realistic time–temperature profiles. Only through such toxicological validation can FBS material‐based 3DFP fulfil their circular‑economy promise without compromising consumer safety.

### 4.4 Nutrient‑Stability and Shelf‑Life Considerations

Early investigations demonstrated that extrusion‐based 3DFP, which typically operates at mild temperatures (20°C–60°C), imposes minimal thermal stress on sensitive micronutrients, allowing most to withstand the printing process largely intact. In strawberry‐based snacks printed with 10%–20% starch, DPPH AC declined by 12% over seven days at ambient temperature, demonstrating partial loss of free‐radical scavenging activity even under mild conditions (Bebek Markovinović et al. 2023). A comparable trend was observed by Severini et al. (2018), in this study 3D‐printed fruit–vegetable snacks stored at 5°C under modified atmosphere lost ∼30% of antioxidant activity and ∼44% of TPC over 8 days, indicating that cold storage alone is insufficient to fully preserve polyphenolic compounds in those newly formed matrices. Aronia melanocarpa (black chokeberry) enriched gellan gels held at 4°C for 14 days maintained their full structural integrity; however, the authors did not measure anthocyanin or polyphenol content at any stage of the process (Zhou et al. 2023). Similarly, orange‐by‐product–fortified apricot gels exhibited enhanced rheological strength, but no data were reported on flavanone stability after freeze‐drying and storage (Molina‐Montero et al. 2023). Mechanistically, nutrient degradation in 3D‐printed constructs could be considered to be primarily driven by oxygen diffusion through the porous lattice, light‐induced oxidation at high surface‐to‐volume ratios, and water‐mediated hydrolysis or Maillard reactions. Preliminary mitigation strategies include lipid‐ or protein‐based microencapsulation, antioxidant co‐formulants, and low‐water‐activity postprint drying have shown promise but require comprehensive kinetic validation. Without standardized shelf‐life protocols that quantify nutrient kinetics across major vitamin, mineral, and bioactive classes, the goal of delivering nutritionally precise, stable 3D‐printed foods will remain unmet.

## 5 Conclusion and Limitations

Over 70 scientific publications highlighted that such underutilized materials could play an important role for the improvement of 3DP performances across a wide spectrum of applications, from packaging, construction, pharmaceutical, biomedical products, and food products. In the case of biocomposite materials for nonfood applications, the incorporation of such food by‐products and food waste can improve several technical performances primarily on the printability and mechanical properties (e.g., beer bagasse, corn and rice husk in PLA filaments, increased tensile strength in copolyester enriched with nut waste) but also on antibacterial properties (e.g., biomedical scaffold made of PLA/crab shell composite), lower carbon emission and other pollutants when incinerated (e.g., for rice husk/PLA composites). In the food sector, the integration of by‐products with other ingredients may improve essential properties of food inks, such as viscosity, shear‐thinning behavior, texture, and mechanical integrity, which are critical for 3DP applications. Fibers, proteins, starch, and bioactives recovered from broccoli, GS, potato‐peel, and salmon‐skin, among others, can significantly enhance the properties of the food inks. In addition, the incorporation of by‐products can increase the nutritional content of 3D‐printed food by up to 22% of micronutrients (e.g., grape pomace in cookies) and up to 45% of proteins (e.g., okara in snacks). Furthermore, the portfolio of the sensory properties can be extended with changes in color, texture, taste, and aroma. Although figures vary by substrate and process, the data collectively indicate that 3D printing with FBS material can routinely cut waste/by‐product disposal volumes by at least 20%, and in some cases by more than half, while simultaneously creating value‐added, nutrient‐fortified foods. Given this the use of upcycled streams extends the opportunity to create nutritional or sensorial customized foods with positive impacts on a more healthy and sustainable diet.

The paper also identifies gaps in current research that deserve further investigation. These include technological challenges such as sophisticated printhead designs and control systems to manage the complex properties of FBS materials, potential safety risks associated with the use of waste‐derived edible 3D‐printed food, regulatory issues, and the need to build consumer trust and acceptance. Most available studies focus on laboratory‐scale formulations, often highlighting the promising nutritional, functional, or structural properties of these materials. However, there is a clear gap between their demonstrated potential and practical integration into scalable 3DP systems. Few studies assess long‐term stability, microbial safety, or regulatory compliance of printed products incorporating these materials, particularly under real‐world conditions. Moreover, while nonfood applications such as packaging, biodegradable composites, and construction materials have been briefly explored, they remain largely conceptual. To fully harness the CE potential of FBS, future work should prioritize (1) formulation optimization for diverse by‐product types, (2) pilot‐scale testing with commercial 3DP platforms, (3) lifecycle and techno‐economic assessments, and (4) cross‐sector application development that bridges food and nonfood domains. Addressing these gaps will be essential to translating the sustainable promise of FBS‐based 3DP into viable industrial practice.

## Author Contributions

**Mohammed A. Bareen**: investigation, writing – original draft, writing – review and editing, methodology, visualization, formal analysis. **Derossi Antonio**: conceptualization, writing – original draft, writing – review and editing, investigation. **Maria G. Corradini**: writing – original draft, writing – review and editing. **Caporizzi Rossella**: writing – original draft, writing – review and editing. **D'Incecco Paolo**: writing – original draft, writing – review and editing, funding acquisition. **Sindaco Marta**: writing – review and editing. **Severini Carla**: writing – review and editing.

## Conflicts of Interest

The authors declare no conflicts of interest.
